# Quantifying the dynamics of the oligomeric transcription factor STAT3 by pair correlation of molecular brightness

**DOI:** 10.1038/ncomms11047

**Published:** 2016-03-24

**Authors:** Elizabeth Hinde, Elvis Pandžić, Zhengmin Yang, Ivan H. W. Ng, David A. Jans, Marie A. Bogoyevitch, Enrico Gratton, Katharina Gaus

**Affiliations:** 1 EMBL Australia Node in Single Molecule Science, School of Medical Sciences, University of New South Wales, Sydney, New South Wales 2052, Australia; 2 ARC Centre of Excellence in Advanced Molecular Imaging, University of New South Wales, Sydney, New South Wales 2052, Australia; 3 Program in Emerging Infectious Diseases, Duke-NUS Medical School, Singapore 169587, Singapore; 4 Nuclear Signalling Lab, Department of Biochemistry and Molecular Biology, Faculty of Biomedical and Psychological Sciences, Monash University, Melbourne, Victoria 3800, Australia; 5 Cell Signalling Research Laboratories, Department of Biochemistry and Molecular Biology, University of Melbourne, Melbourne, Victoria 3010, Australia; 6 Laboratory for Fluorescence Dynamics, Department of Biomedical Engineering, University of California, Irvine, California 92697-2715, USA

## Abstract

Oligomerization of transcription factors controls their translocation into the nucleus and DNA-binding activity. Here we present a fluorescence microscopy analysis termed pCOMB (pair correlation of molecular brightness) that tracks the mobility of different oligomeric species within live cell nuclear architecture. pCOMB amplifies the signal from the brightest species present and filters the dynamics of the extracted oligomeric population based on arrival time between two locations. We use this method to demonstrate a dependence of signal transducer and activator of transcription 3 (STAT3) mobility on oligomeric state. We find that on entering the nucleus STAT3 dimers must first bind DNA to form STAT3 tetramers, which are also DNA-bound but exhibit a different mobility signature. Examining the dimer-to-tetramer transition by a cross-pair correlation analysis (cpCOMB) reveals that chromatin accessibility modulates STAT3 tetramer formation. Thus, the pCOMB approach is suitable for mapping the impact oligomerization on transcription factor dynamics.

## Abstract (web summary)

Oligomerisation of transcription factors regulates their translocation into the nucleus, DNA binding affinity and sequence specificity. Here, Hinde *et al*. present a microscopy analysis, pair correlation of molecular brightness, that tracks the molecular mobility of different oligomeric species in the same cell.

## Graphical abstract

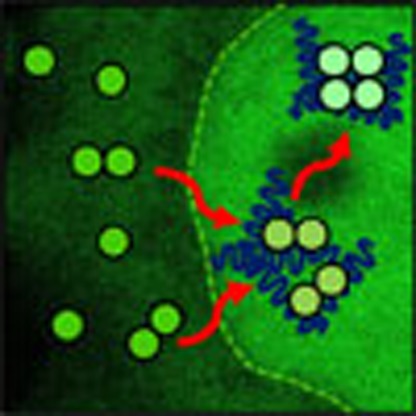

Transcription factors are DNA-binding proteins that affect the rate of transcriptional initiation and employ oligomerization to define discrete regulatory pathways^1^. Homo- and hetero-oligomerization of a transcription factor can change DNA-binding affinity, alter sequence specificity and vary the modes of transcriptional regulation. In fact, exchange of a single component within a transcription factor complex can transform it from one that activates transcription to one that represses gene expression^1^. For example, the signal transducers and activators of transcription (STATs) are a class of transcription factor that transmit signals from the plasma membrane to target genes in the nucleus, and through reversible formation of homo- or hetero-dimers, modulate the kinetics of nuclear trafficking to turn on or off gene expression^2^,^3^,^4^. Specifically, STAT3 monomers form latent dimers that shuttle between the nucleus and cytoplasm, and on phosphorylation bind consensus DNA targets to induce gene expression^4^,^5^. It is also known that the nuclear dimer population can further interact to form STAT3 tetramers on adjacent gamma interferon activation (GAS) elements, which bend the DNA into a conformation that amplifies or represses STAT3 dimer-regulated gene expression^6^,^7^,^8^. To decipher how these oligomerization events control transcription factor nuclear access, inform target search strategies and confer DNA-binding activity requires a method that can quantify the molecular mobility of each oligomeric state. Thus, here we set out to establish and apply an imaging-based approach that could quantify the molecular mobility of the different STAT3 oligomeric species and thus map STAT3 dimer versus tetramer DNA binding in a live cell.

Förster resonance energy transfer (FRET)^9^,^10^,^11^, fluorescence correlation spectroscopy (FCS)^12^,^13^,^14^,^15^,^16^,^17^ and the photon-counting histogram (PCH)^18^,^19^,^20^ have to date been used to detect self-association of fluorescent fusion proteins^21^. Fluctuation-based methods of analysis such as FCS or PCH extract the oligomeric state of a protein from the brightness of the fluorescent complex^22^. This requires that the number of molecules in a given volume is determined first by a moment-based analysis, and then the brightness of the fluorescent molecules is obtained by a ratio of the average intensity to the number of molecules. Moment analysis was originally proposed by Qian and Elson^23^ for molecules in solution and more recently, the concept was extended from a single-point measurement to a confocal frame acquisition, termed Number and Brightness (N&B) analysis^24^,^25^. N&B derives a spatial map of a protein's oligomeric state within a live cell, with pixel-level resolution.

A limitation of moment analysis in general is that multiple species of different brightness cannot be resolved using only the first and second moments of a brightness distribution. Higher moments are needed and this demands acquisition of a much larger data set. For example, while PCH analysis can resolve multiple species in a single point by considering the entire distribution of photon counts collected, it is computationally too laborious to apply the PCH analysis to all pixels within an image^24^. Thus, with the current brightness analyses one can either (1) measure the components of an oligomeric distribution in a single location as a function of time or (2) assemble a spatial map of the average oligomeric state in each pixel at a particular time point. Because of this limitation, it is currently not possible to track molecules as a function of their oligomeric state. For example, it is not known how STAT3 dimerization changes the kinetics of STAT3 nucleocytoplasmic transport or whether STAT3 tetramer formation is required for, or is a consequence of, DNA binding^4^,^6^. One could distinguish between these different scenarios if one could measure the mobility of monomers, dimers and tetramers independently.

Spatiotemporal correlation spectroscopy can reveal the diffusive route of a population of molecules in real time and across cellular boundaries^21^,^26^. For example, pair correlation analysis is a method that spatially correlates fluctuations in fluorescence intensity acquired along a confocal line scan, and we recently demonstrated that this method can be used to quantify the impact of chromatin organization on nuclear protein translocation^27^,^28^,^29^. However, this method of analysis, when applied to intensity fluctuations, indicates only the number of molecules that perform a transit and does not discriminate transport based on oligomeric state. Thus, we propose to perform pair correlation analysis directly on brightness fluctuations (a property that can be translated to oligomeric state) and independently track the mobility of different oligomeric species across cellular compartments. That is, instead of performing a higher-order moment or correlation analysis to resolve the dynamics of a heterogeneous oligomeric population within an observation volume^30^,^31^,^32^, we introduce a spatial component to the correlation function that selectively amplifies the brightest species and filters the different-sized oligomers within this population, based on their distinct arrival times between two locations.

Here we call this method of analysis pair correlation of molecular brightness (pCOMB), and demonstrate the capacity of this approach to quantify the impact transcription factor oligomerization has on nuclear transport and DNA-binding dynamics. From the application of pCOMB to wild-type and mutant STAT3 fluorescent proteins in live HeLa cells we discovered that phosphorylated STAT3 dimers needed to bind DNA before forming tetramers, and from extension to a cross-pair correlation analysis^27^ (cross-pair correlation analysis of molecular brightness; cpCOMB), that the dimer-to-tetramer transition is regulated by chromatin accessibility and cytokine stimulation.

## Results

### Analysis of molecular brightness fluctuations

**Figure 1:**
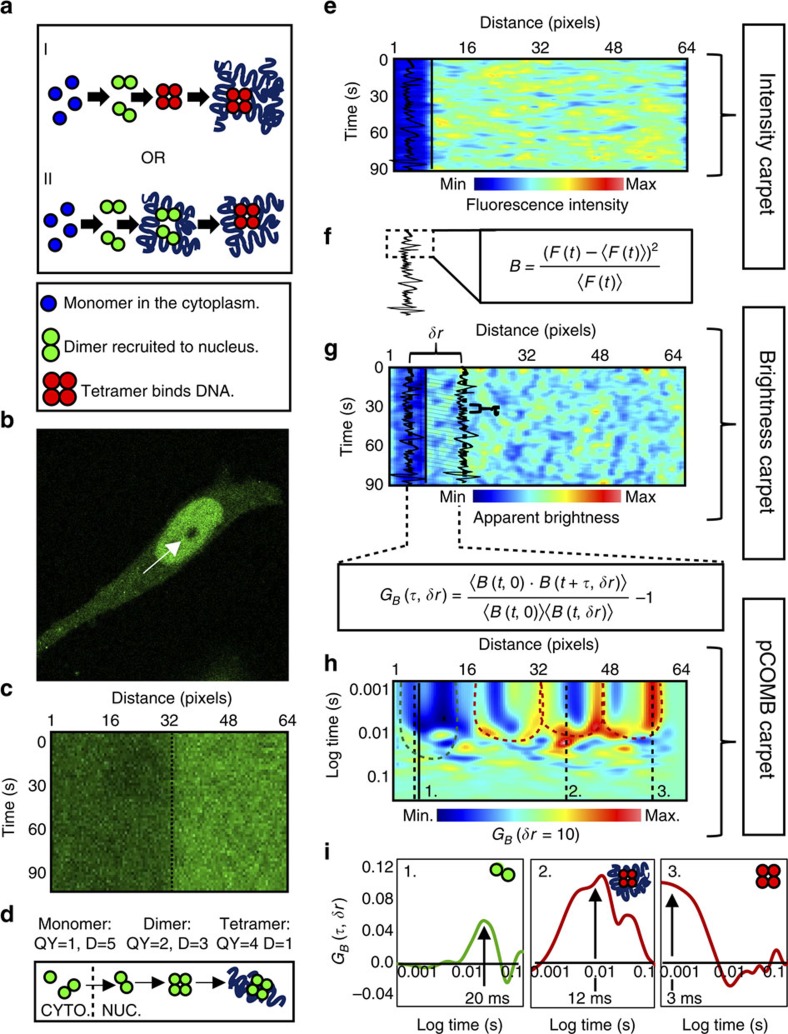
Tracking protein oligomerization in live cells by pCOMB. (**a**) pCOMB is designed to distinguish the following two scenarios: STAT3 dimers form tetramers during target search (scenario I) or after binding to the target DNA sequence (scenario II). (**b**) Intensity image of a HeLa cell expressing STAT3-GFP. Location of the confocal line scan (64 pixels, white arrow) from the cytoplasm to nucleus is indicated. (**c**) Kymograph of STAT3-GFP intensity fluctuations along line scan. Black line indicates the cytoplasm–nucleus boundary. (**d**) Simulation of scenario I: monomers (QY *D*=5 μm^2^ s^−1^, density=50%) and dimers (*D*=3 μm^2^ s^−1^, density=40%) diffused in the cytoplasmic compartment. Only dimers could translocate to the nucleus where tetramers (*D*=1 μm^2^ s^−1^, density=10%) reversibly bound to DNA (unbinding probability 0.1). (**e**) Simulated intensity carpet along a 64-pixel line. (**f**) Each intensity fluctuation in **e** was transformed into a brightness fluctuation by calculating a moving average of the apparent brightness (*B*). (**g**) The resulting brightness carpet was the data format on which pair correlation analysis (*G*_*B*_(*τ*, *δr*)) was performed. (**h**,**i**) In the final pCOMB carpet (*δr*=10), the pseudocoloured correlation amplitude reflects the oligomeric state and the delay time (*y* axis) reflects the mobility of the oligomeric state (**h**). (**i**) From specific columns in the pCOMB carpet (dashed lines at position 1, 2 and 3 in **h**), the dimer translocation into the nucleus (green dotted arc in **h**) and tetramer interaction with chromatin regions (red dotted arcs in **h**) was extracted: (1) dimers transited into the nucleus with a maximum transit time of ∼20 ms, (2) and (3) tetramers exhibited two distinct transit times of 12 and 3 ms, corresponding to DNA-unbound and -bound tetramers, respectively (**i**). CTYO, cytoplasm; D, diffusion coefficient; Max, maximum; Min, minimum; NUC, nucleus; QY, quantum yield.

The current state of the literature allows for two scenarios: either STAT3 dimers in the nucleus first form tetramers that subsequently bind DNA (scenario I in Fig. 1a) or STAT3 dimers bind DNA before forming tetramers (scenario II in Fig. 1a). Pair correlation analysis of STAT3-GFP intensity fluctuations along a line scan (Fig. 1b,c) cannot resolve this issue since this analysis cannot distinguish the oligomeric state of GFP (green fluorescent protein) fusion proteins. Conversely, N&B analysis could identify the dominant oligomeric species in each pixel but cannot report the molecular mobility of that species and hence distinguish freely diffusive STAT3 tetramers from DNA-bound STAT3 oligomers. To overcome these difficulties and in essence combine the analyses, we introduce a spatial component to the moment analysis, which filters the arrival time of the differently bright molecules between two locations. This new analysis returns the transit time for a given oligomeric species, that is, the time a given species takes to diffuse between the two locations. As transcription factors bound to DNA have limited mobility^33^, this approach could distinguish between the two scenarios in Fig. 1a.

The method is built on a confocal intensity line-scan acquisition across a live cell that expresses a fluorescently labelled transcription factor (Fig. 1b,c). We simulated the scenario I in Fig. 1a, where tetramers could diffuse freely and bind to DNA in the nucleus (Fig. 1d, see Methods for simulation details). The resulting intensity fluctuations (Fig. 1e) were transformed into brightness fluctuations (Fig. 1f), and the pair correlation analysis was then performed on the brightness fluctuations along the line scan (Fig. 1g), at a distance (*δr*) which tested molecular flow across the nuclear envelope and DNA-binding regions. This yielded a pCOMB carpet (Fig. 1h) in which the pseudocolour amplitude reflects the oligomeric state of the protein and the transit time the mobility in that pixel (dashed green arc shows dimers traversing the nuclear envelope and dashed red arcs show tetramers transiently binding DNA). For example, extraction of specific transits from the pCOMB carpet (columns 1, 2 and 3 in Fig. 1h) found dimers and tetramers to be easily distinguishable based on pCOMB correlation amplitude (green and red for dimers and tetramers, respectively, in Fig. 1h,i). As expected, we found the two different transit times for tetramers (12 and 3 ms), which corresponded to tetramers bound and not bound to DNA.

**Figure 2:**
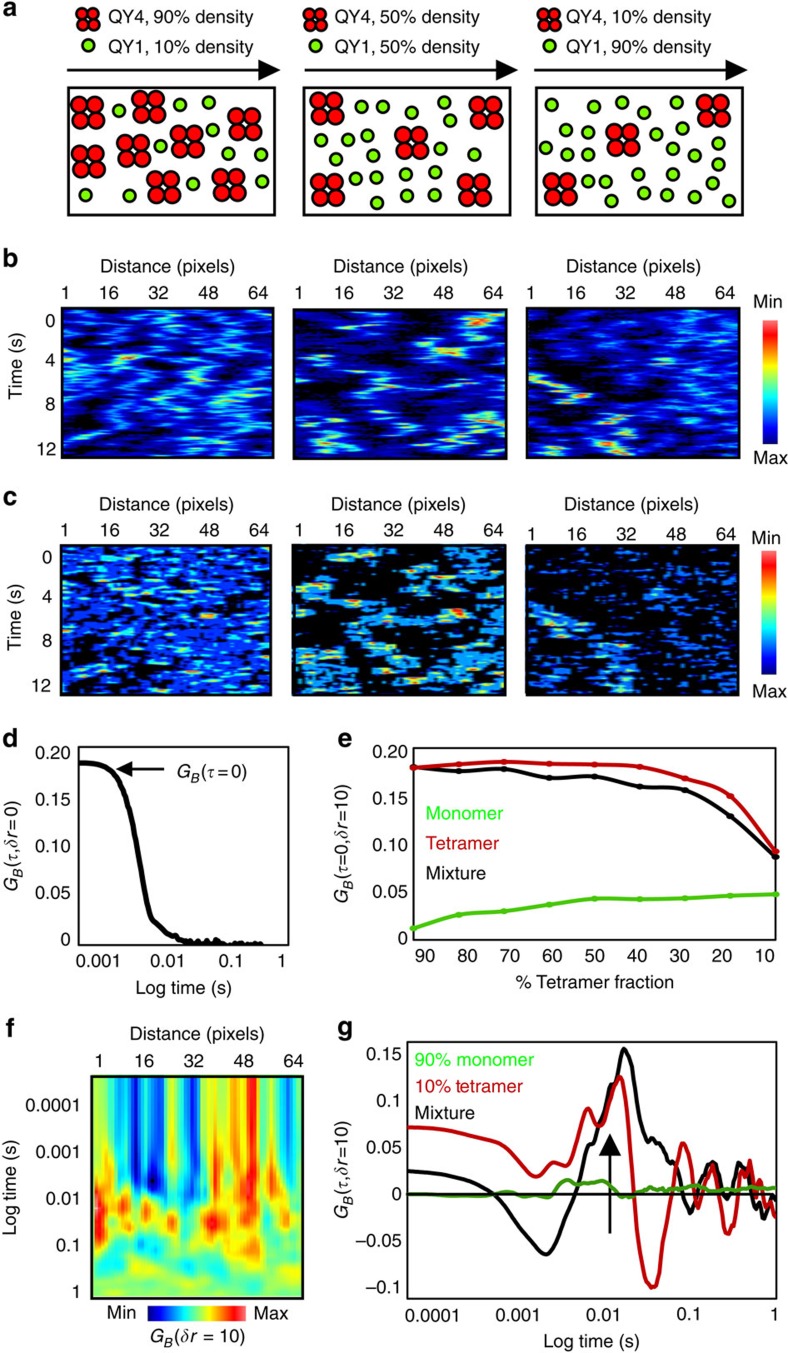
Higher-order oligomers are amplified in the pCOMB analysis. (**a**–**g**) Simulation of various ratio of tetramers to monomers (both diffusing at 1 μm^2^ s^−1^; ratios 9:1, 5:5 and 1:9). (**b**) Intensity carpets of the three selected simulations. (**c**) Brightness carpets of the three simulations. (**d**) Correlation analysis of a brightness fluctuation with itself (*δr*=0) resulted in an autocorrelation function, where *G*_*B*_ at *τ*=0 was proportional to the weighted square of the brightness of the molecules. (**e**) *G*_*B*_(0) value derived from autocorrelation analysis of the individual components (tetramer in red and monomer in green) and mixed population (black line) for various tetramer factions. (**f**) pCOMB analysis (*δr*=10) of the simulation, which contained 10% tetramer in the presence of 90% monomer. (**g**) Comparison of the average pair correlation profile of the individual components (tetramer in red and monomer in green) and mixed population (black line) of 10% tetramer and 90% monomer. Black arrow highlights average transit time. Max, maximum; Min, minimum; QY, quantum yield.

By selective amplification of the signal from the highest-order oligomer complex, the pCOMB analysis has the potential to reveal the mobility of different oligomeric species of the same transcription factor, even though the fraction of transcription factor that actively regulates gene expression at the target site, and thus is bound to DNA at any given time, may be relatively low (simulated in Fig. 1d as 10%). To demonstrate the rules of analysis when spatially cross-correlating brightness fluctuations, we simulated line-scan data for different heterogeneous oligomeric populations. The key concept behind this method is that the spatial cross-correlation function preferentially amplifies the signal from the brightest species and therefore can reveal higher-order oligomer translocation in the presence of a dominating population of monomers. To illustrate this, we first simulated a line scan for a heterogeneous population of monomers (quantum yield=1) and tetramers (quantum yield=4) both diffusing with a coefficient of 1 μm^2^ s^−1^, and then varied the density of each population (Fig. 2a). Next, we transformed the intensity carpets (Fig. 2b) into brightness carpets (Fig. 2c) by carrying out a moment analysis on the simulated intensity fluctuations (see Methods for details). The brightness transformation enhanced the differences in oligomer density between the three examples.

To first demonstrate how a correlation function preferentially amplifies the signal from few higher-order oligomers in the presence of monomers, we performed an autocorrelation analysis (*δr*=0) on each brightness carpet (Fig. 2d) and plotted the correlation amplitude at *τ*=0 as a function of the percentage tetramer present (Fig. 2e). This analysis revealed that first, the *G*_*B*_(*τ*=0, *δr*=0) value of the heterogeneous population (black line in Fig. 2e) was almost equal to the *G*_*B*_(*τ*=0, *δr*=0) of the tetrameric population (red line in Fig. 2e), even when tetramers constitute only 10% of the population, and second, the *G*_*B*_(*τ*=0, *δr*=0) value of the monomeric population (green line in Fig. 2e) remains constant when monomers constitute 40–90% of the population. The implication of these two effects on the pair correlation analysis of brightness fluctuations (*δr*>0) is that the translocation path of a small population of higher-order oligomer (10% in Fig. 2f) could be extracted and differentiated from a dominating monomer translocation (black arrow in Fig. 2g) when the *G*(0) value for monomeric molecular flow at a given *δr* is known. It should be noted that the number of subunits within an oligomeric complex determines the accuracy with which this method can resolve this complex's translocation path. Thus, while detection of even 1–10% tetramer in the presence of monomer was relatively easily resolved based on correlation amplitude (black versus green curves in Supplementary Fig. 1a), the brightness difference between monomers and dimers was less significant, and therefore distinguishing their diffusive routes based on correlation amplitude may be less accurate at these low percentages (Supplementary Fig. 1b). Similarly to fluorescence intensity-based correlation analysis, cross correlation of molecular brightness fluctuations results in autocorrelation profiles with a decay indicative of the rate of diffusion (Supplementary Fig. 1c) and pair correlation profiles with a peak maximum indicative of the resulting translocation time (Supplementary Fig. 1d).

### pCOMB analysis tracks oligomer translocation

**Figure 3:**
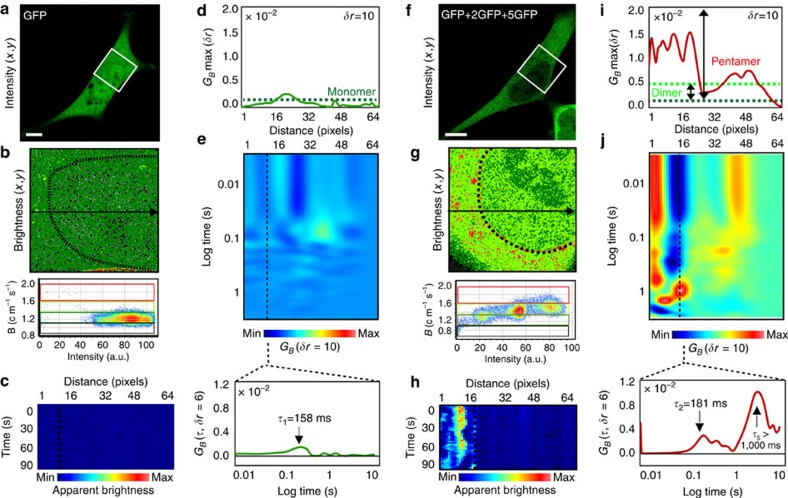
Calibration of pCOMB. (**a**) HeLa cell transfected with monomeric GFP. Scale bar, 5 μm. (**b**) Brightness analysis of GFP in the region highlighted in **a**. Brightness map (top) is pseudocoloured according to coloured cursors placed over the brightness distribution (bottom). Dark green pixels represent monomers. Nuclear boundary is indicated with black dotted line. (**c**) Brightness carpet derived from the line scan indicated in **b**. (**d**) Pair correlation maximum amplitude (*G*_*B*_max) in each pixel along line scan. Dashed line represents the average *G*_*B*_max and this value was used to calibrate higher-order oligomer translocation. (**e**) pCOMB carpet. Dashed line indicates nuclear envelope. Insert: pair correlation profile for GFP monomers entering the nucleus with a transit time of ∼158 ms. (**f**) HeLa cell transfected with monomeric, dimeric and pentameric GFP. (**g**) Brightness analysis of GFP, 2GFP and 5GFP in region highlighted in **f**. Brightness map (top) is pseudocoloured according to coloured cursors placed over the brightness distribution (bottom). Dark green pixels represent monomers, light green pixels dimers and red pixels pentamers. (**h**) Brightness carpet derived from line scan shown in **g**. (**i**) Pair correlation maximum amplitude in each pixel along line scan, overlaid with the pair correlation amplitude calibrated for monomeric GFP and the extrapolated pair correlation amplitude for dimeric or pentameric GFP. (**j**) pCOMB carpet. Dashed line indicates nuclear envelope. Insert: pair correlation profile for GFP dimers and pentamers entering the nucleus reveals a transit time of ∼181 and >1,000 ms, respectively. Max, maximum; Min, minimum.

To experimentally confirm the insights gained from simulation, we next applied the analysis to different GFP constructs of known stoichiometry (monomer, dimer and pentamer)^34^. Because the link between correlation amplitude and oligomeric state relies on careful calibration of the monomeric brightness, HeLa cells were transiently transfected with monomeric GFP (Fig. 3a–e) and compared with cells transiently transfected with a combination of GFP, 2GFP and 5GFP at equal concentrations of DNA (Fig. 3f–j). Brightness analysis of GFP within a selected region (Fig. 3a) showed a homogenous population of GFP monomers throughout the nucleus and cytoplasm (green pixels in Fig. 3b). Pair correlation analysis of GFP brightness fluctuations along a line scan (Fig. 3c) that went across these two intracellular compartments resulted in an average maximum correlation amplitude of 0.001±0.0005 (Fig. 3d), irrespective of the transit time recorded in the pCOMB carpet (Fig. 3e). This value was used to set the amplitude for GFP monomer translocation in the cell that co-expressed GFP dimers and pentamers (Fig. 3f-h), where higher correlation amplitude values (0.001–0.016, Fig. 3i) indicated the presence of oligomers. We could now assign these higher correlation amplitudes to the different GFP oligomers present, with dimers expected to fall within the range of 0.001–0.004 (2^2^ × 0.001=0.004) and GFP pentamers in the range of 0.001–0.025 (5^5^ × 0.001=0.025). On the basis of this assignment, the pCOMB carpet (Fig. 3j) revealed different transit times for monomers versus oligomers. For example, while monomers were found to translocate the nuclear envelope within ∼158 ms (insert in Fig. 3e), dimers and pentamers were found to the enter the nucleus with a transit time of ∼181 and >1,000 ms, respectively (insert in Fig. 3j). The difference in transit time detected for monomeric GFP compared with dimeric or pentameric GFP was expected, given the size restrictions imposed by the nuclear pore complex^35^,^36^,^37^.

Thus, we could experimentally extract and measure the diffusive route of GFP oligomers in the presence of GFP monomers, and concluded that the ‘rules' of pair correlation brightness carpets are (1) the amplitude is proportional to the weighted square of the molecular brightness of the fluorescent molecules and (2) the peak maximum is indicative of the translocation time. It should be noted that while the translocation path of the higher-order oligomer can definitively be assigned based on the correlation amplitude, a single-channel acquisition may have difficulties distinguishing smaller oligomers when their delay times are similar (which was not the case in Fig. 3j).

### STAT3 dynamics are regulated by oligomerization

**Figure 4:**
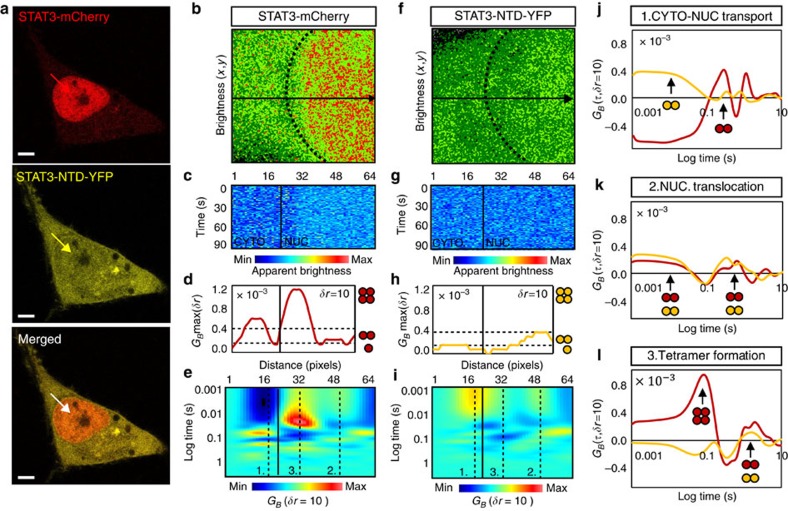
pCOMB analysis of STAT3 translocation as a function of oligomeric state. (**a**) HeLa cell transfected with STAT3-mCherry and STAT3-NTD-YFP after 15-min stimulation with 10 nM oncostatin M. Region of interest for brightness and pCOMB analysis is indicated by the arrow. Scale bar, 5 μm. (**b**) Brightness analysis of STAT3-mCherry: dark green pixels represent monomers, light green pixels dimers and red pixels tetramers. STAT3-mCHerry brightness distribution and monomer calibration are presented in Supplementary Fig. 3a–d. (**c**–**e**) pCOMB analysis: brightness carpet (**c**); pair correlation maximum amplitude *G*_*B*_max (**d**); and pCOMB carpet (**e**) for STAT3-mCherry. *G*_*B*_max was calibrated with respect to monomeric mCherry in Supplementary Fig. 3e–f. (**f**) Brightness analysis of STAT3-NTD-YFP (brightness distribution and monomer calibration are presented in Supplementary Fig. 4a–d). (**g**–**i**) pCOMB analysis: brightness carpet (**g**); pair correlation maximum amplitude *G*_*B*_max (**h**); and pCOMB carpet (**i**) for STAT3-NTD-YFP. *G*_*B*_max was calibrated to monomeric YFP in Supplementary Fig. 4e,f. In **e** and **i**, pCOMB carpet was pseudocoloured from monomer (blue) to highest-order oligomer (red). (**j**–**l**) pCOMB profiles for STAT3-mCherry (red) and STAT3-NTD-YFP (yellow) translocation across the nuclear envelope (**j**, position 1 in **e**,**i**), intra-nuclear dimer translocation (**k**, position 2 in **e**,**i**) and intra-nuclear tetramer formation (**l**, position 3 in **e**,**i**). CTYO, cytoplasm; Max, maximum; Min, minimum; NUC, nucleus.

Next we investigated whether the translocation dynamics of STAT3 depends on the oligomeric state of the transcription factor and thus tested whether scenario I or II (Fig. 1a) applied *in vivo*. To experimentally verify the outcomes of the pCOMB analysis, we compared wild-type STAT3-mCherry with a STAT3 mutant lacking the N-terminal domain (STAT3-NTD-YFP) in the same HeLa cell. The N-terminal deletion mutant^5^ was previously shown to (i) inhibit latent dimer formation in the cytoplasm, resulting in a predominately monomeric cytoplasmic population in resting cells^4^ and (ii) impaired phosphorylated dimer–dimer interaction in the nucleus, which prevents tetramer formation during transcriptional activation^8^. The compromised oligomeric subcellular distribution of this mutant was verified by a brightness analysis of STAT3-NTD-YFP before and after stimulation, with the cytokine oncostatin M (10 nM, 15 min, Supplementary Fig. 2). Thus acquisition of two-colour line-scan data of STAT3-mCherry and STAT3-NTD-YFP in a stimulated cell (Fig. 4a) not only acted as an internal control for pCOMB detection of STAT3 oligomerization but also allowed us to identify STAT3 activity, that is, where STAT3-mCherry was visible and tetrameric in the nucleus. The brightness map derived for STAT3-mCherry detected STAT3 monomers and dimers in the cytoplasm, and STAT3 dimers and tetramers in the nucleus (Fig. 4b and Supplementary Fig. 3a–d). pCOMB analysis of STAT3-mCherry brightness fluctuations along the selected line scan (Fig. 4c) resulted in correlation amplitudes (Fig. 4d and Supplementary Fig. 3e–f) and corresponding transit times (Fig. 4e) that demonstrated STAT3 monomers and dimers traversed the nuclear envelope on a timescale of ∼279 ms (position 1 in pCOMB carpet), explored the nucleus on a fast timescale of ∼12 ms and slow timescale of ∼583 ms (position 2 in pCOMB carpet), and formed tetramers that diffused on a slow timescale of ∼203 ms (position 3 in pCOMB carpet). The lack of fast timescale diffusion observed for the tetrameric population suggests that only DNA-bound STAT3-mCherry tetramers existed and therefore scenario II in Fig. 1a applied.

The brightness map derived for STAT3-NTD-YFP reconfirmed that deletion of the N-terminal domain inhibited cytoplasmic dimer formation and nuclear tetramer formation (Fig. 4f and Supplementary Fig. 4a–d). Comparison of the correlation amplitudes and corresponding transit times derived from pCOMB analysis of STAT3-NTD-YFP (Fig. 4g-i and Supplementary Fig. 4e–f) with those of STAT3-mCherry revealed that the few STAT3-NTD-YFP dimers (that were hardly detectable in the brightness analysis in Fig. 4f–g) diffused across the nuclear envelope very rapidly in 14 ms (Fig. 4j) had a similar molecular mobility within the nucleus as wild-type STAT3-mCherry (fast timescale of ∼10 ms and slow timescale of ∼362 ms Fig. 4k) but failed to form tetramers (Fig. 4l). Taken together, these experiments highlighted the sensitivity of the pCOMB method, which in a single cell detected the formation of a population of transiently bound STAT3-mCherry tetramers and a small population of STAT3-NTD-YFP dimers entering the nucleus on a fast timescale. This suggests that the STAT3 N-terminal domain was not required for nuclear translocation or DNA binding but for DNA-bound dimer–dimer interactions and/or tetramer formation, schematically described as scenario II in Fig. 1a.

### STAT3 DNA binding is necessary for tetramerization

**Figure 5:**
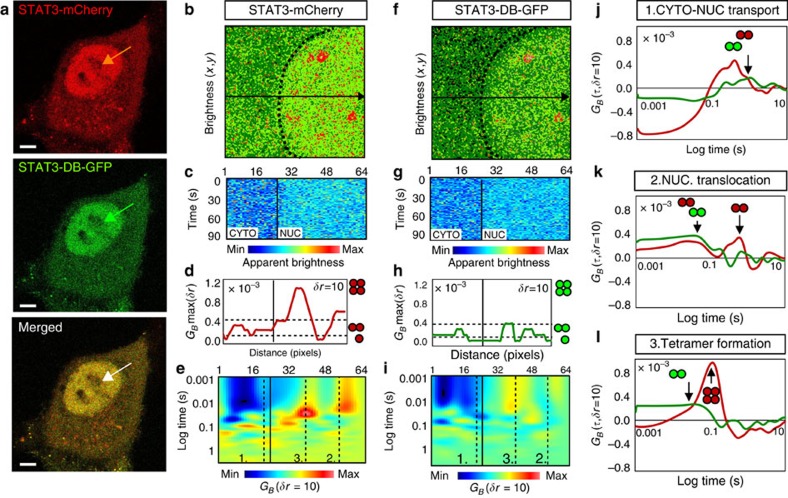
pCOMB analysis of STAT3 DNA binding as a function of oligomeric state. (**a**) HeLa cell transfected with STAT3-mCherry and STAT3-DB-GFP after 15-min stimulation with 10 nM oncostatin M. Region of interest for brightness and pCOMB analysis is indicated by the arrow. Scale bar, 5 μm. (**b**) Brightness analysis of STAT3-mCherry: dark green pixels represent monomers, light green pixels dimers and red pixels tetramer (mCherry monomer calibration presented in Supplementary Fig. 3a–d). (**c**–**e**) pCOMB analysis: brightness carpet (**c**); pair correlation maximum amplitude *G*_*B*_max (**d**); and pCOMB carpet (**e**) for STAT3-mCherry. *G*_*B*_max was calibrated with respect to monomeric mCherry in Supplementary Fig. 3e–f. (**f**) Brightness analysis of STAT3-DB-GFP (brightness distribution and monomer calibration are presented in Supplementary Fig. 6a–d). (**g**–**i**) pCOMB analysis: brightness carpet (**g**); pair correlation maximum amplitude *G*_*B*_max (**h**); and pCOMB carpet (**i**) for STAT3-DB-GFP. *G*_*B*_max was calibrated to monomeric GFP in Supplementary Fig. 6e–f. In **e** and **i**, pCOMB carpet was pseudocoloured from monomer (blue) to highest-order oligomer (red). (**j**–**l**) pCOMB profiles for STAT3-mCherry (red) and STAT3-DB-GFP (green) translocation across the nuclear envelope (**j**, position 1 in **e**,**i**), intra-nuclear dimer translocation (**k**, position 2 in **e**,**i**) and intra-nuclear tetramer formation (**l**, position 3 in **e**,**i**). CTYO, cytoplasm; Max, maximum; Min, minimum; NUC, nucleus.

Our results so far suggest that DNA-bound dimers are required for tetramer formation, and STAT3 tetramers do not diffuse freely in oncostatin-stimulated cells. We next examined a DNA-binding mutant (STAT3-DB-GFP) that contained a double mutation within its DNA-binding domain (Glu-434 and Glu-435 residues replaced by alanines)^38^,^39^. We again compared wild-type STAT3-mCherry with STAT3-DB-GFP in cells stimulated with oncostatin M (10 nM, 15 min, Supplementary Fig. 5), which induced nuclear accumulation of STAT3 irrespective of the DNA-binding ability (Fig. 5a). As expected, brightness (Fig. 5b) and pCOMB (Fig. 5c–e) analysis of STAT3-mCherry recovered an oligomeric subcellular distribution and translocation path that were similar to those seen in Fig. 4. The brightness map derived for STAT3-DB-GFP indicated a reduction in nuclear dimers and tetramers (Fig. 5f and Supplementary Fig. 6a–d). pCOMB and brightness analysis of STAT3-DB-GFP (Fig. 5g–i and Supplementary Fig. 6e,f) revealed that the loss of DNA-binding capacity did not impact on STAT3 dimer translocation to the nucleus, which occurs on a timescale of ∼378–551 ms (Fig. 5j). In contrast, while for wild-type STAT3-mCherry dimers, two transit times were observed within the nucleus (∼31 and ∼651 ms), STAT3-DB-GFP only exhibited the fast translocation time (∼54 ms, Fig. 5k). This suggests that the slow transit time (651 ms) of wild-type STAT3 dimers is due to transient interactions with DNA. Importantly, no STAT3-DB-GFP tetramers were observed (Fig. 5l), strongly indicating that DNA binding was required and preceded tetramer formation.

Given that both STAT3 DNA binding of GAS elements and tetramer formation are reported to require tyrosine phosphorylation^8^, we repeated the experiment with a STAT3 phosphorylation mutant in which tyrosine 705 is substituted by phenylalanine (Y705F)^40^,^41^. We performed brightness (Supplementary Fig. 7a,b) and pCOMB analysis of STAT3-mCherry and STAT3-YF-GFP in an oncostatin M-stimulated cell (Supplementary Fig. 7c–f). The lack of STAT3-YF-GFP tetramer formation verified that STAT3 tetramer formation was phosphorylation dependent. Similarly, the observation that STAT3-YF-GFP dimers only had a fast transit time strongly indicated that the wild-type STAT3 dimer population that exhibited slow nuclear transit times due to DNA interactions was indeed phosphorylated (Supplementary Fig. 7e). Thus, in conclusion we show that while the DNA-binding domain of STAT3 was not critical for the recruitment of dimers to the nucleus, the nuclear population of phosphorylated STAT3 dimers must bind DNA for STAT3 tetramer formation.

### Cross pCOMB independently tracks dimers and tetramers

**Figure 6:**
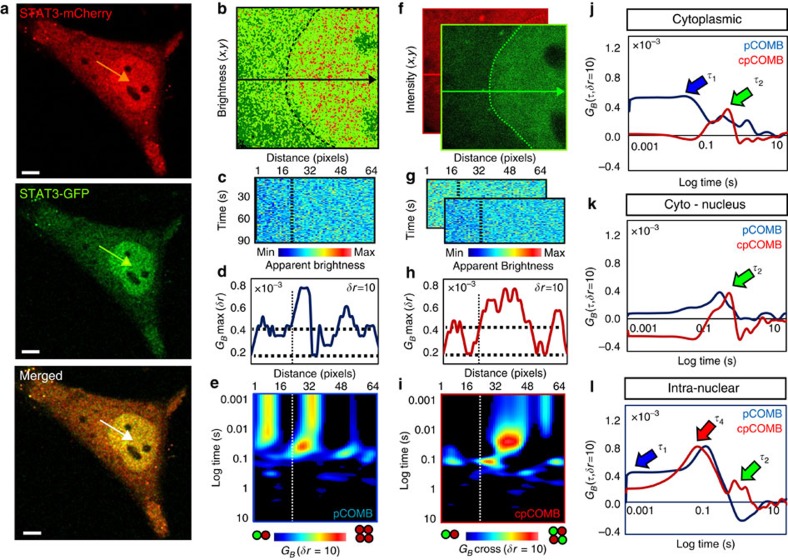
Cross-pcCOMB analysis of STAT3 homo-oligomers. (**a**) HeLa cell transfected with STAT3-GFP and STAT3-mCherry after 15-min stimulation with 10 nM oncostatin M, with the position of two-colour line scan superimposed. Scale bar, 5 μm. (**b**) Brightness analysis of STAT3-GFP within the plane in which the line scan was acquired: dark green pixels represent monomers, light green pixels dimers and red pixels tetramer. (**c**–**e**) pCOMB analysis: brightness carpet (**c**); pair correlation maximum amplitude *G*_*B*_max (**d**); and pCOMB carpet (**e**), pseudocoloured from monomer (blue) to highest-order oligomer (red) for STAT3-GFP. (**f**) STAT3-GFP and STAT3-mCherry fluorescence intensity within the plane in which the line scan was acquired. (**g**) Brightness carpets for STAT3-GFP and STAT3-mCherry that were used for cross pCOMB analysis. (**h**) Cross-pair correlation maximum amplitude along the line scan, calibrated with respect to the expected cross-pair correlation amplitude for a GFP-mCherry heterodimer. (**i**) cpCOMB carpet pseudocoloured from heterodimer (blue) to the highest-order hetero-oligomer (red). (**j**–**l**) pCOMB and cpCOMB profiles for cytoplasmic translocation (**j**), across the nuclear envelope (**k**) and for intra-nuclear translocation (**l**). Blue, green and red arrows indicate transit time for monomer, dimer and tetramer, respectively. CTYO, cytoplasm; Max, maximum; Min, minimum.

A weakness of the pCOMB analysis is that monomers and dimers can be difficult to distinguish. Given the importance of dimerization for many transcription factors and other signalling proteins, we establish cpCOMB, using again two-channel acquisitions. When two subunits are fluorescently labelled with two different fluorophores, cpCOMB only tracks heterocomplexes. By expressing STAT3-GFP and STAT3-mCherry in the same cell, cpCOMB enables the translocation path of STAT3 dimers to be easily distinguished from that of STAT3 tetramers and monomers. This is because the cross-correlation function further amplifies the highest-order oligomer whilst removing the contribution of monomers (Supplementary Fig. 8a–c). To experimentally test this analysis, we acquired a simultaneous two-colour line-scan experiment across STAT3-GFP and STAT3-mCherry in a stimulated HeLa cell (Fig. 6a). As expected, brightness (Fig. 6b) and pCOMB analysis (Fig. 6c–e) of the STAT3-GFP channel recover an oligomeric subcellular distribution and translocation path that were in agreement with the dynamics detected in Figs 4 and 5. Next, we performed cpCOMB analysis between the STAT3-GFP and STAT3-mCherry channels (Fig. 6f,g) and plotted the cross-correlation amplitudes (Fig. 6h) and corresponding cpCOMB carpet (Fig. 6i). Comparison of the pCOMB profile (with monomers) with the cpCOMB profile (without monomers) demonstrated the following: (i) STAT3 monomers and dimers translocate the cytoplasm on a timescale of ∼30 ms (blue arrow) and ∼130 ms (green arrow), respectively (Fig. 6j); (ii) STAT3 dimers crossed the nuclear envelope on a timescale of ∼200 ms (green arrow; Fig. 6k); and (iii) through further amplification of the highest-order oligomer present (and in agreement with Figs 4l and 5l), STAT3 dimers were found to give rise to fast and slow transit (∼69 and ∼719 ms), whereas the tetramers only exhibited delayed mobility (∼279 ms). Thus, the cpCOMB analysis makes dimer detection much more straightforward and confirmed the assignment of these oligomers in previous pCOMB measurements. Artefact due to spectral crosstalk between the two channels of the acquisition (Supplementary Fig. 8d,e) or FRET between STAT3-GFP and STAT3-mCherry on STAT3 oligomerization (Supplementary Fig. 8f) was found to be negligible.

### Modulation of the STAT3 dimer-to-tetramer transition

**Figure 7:**
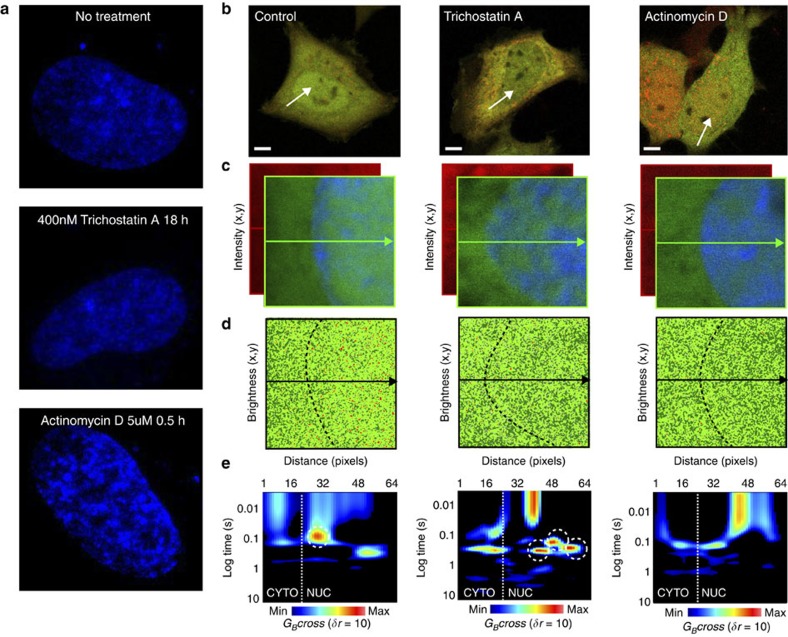
Chromatin accessibility regulated STAT3 dimer-to-tetramer transition (cpCOMB analysis). (**a**) Chromatin organization in HeLa cells stained with Hoechst 33342 after no treatment, 18 h after addition of 400 nM of trichostatin A (loosened chromatin) and 30 min after addition of 5 μg ml^−1^ of actinomycin D (compacted chromatin). (**b**) Merged STAT3-GFP and STAT3-mCherry intensity images of control, trichostatin A- and actinomycin D-treated live HeLa cells. Scale bar, 5 μm. (**c**) STAT3-GFP and STAT3-mCherry fluorescence intensity within the plane in which the line scan was acquired. The nuclear boundary is indicated by the Hoechst 33342 stain. (**d**) Pseudocoloured brightness maps for STAT3-GFP in control, trichostatin A- and actinomycin D-treated HeLa cells. Dark green pixels represent monomers, light green pixels dimers and red pixels tetramer. (**e**) cpCOMB carpets in control, trichostatin A- and actinomycin D-treated live HeLa cells. White dashed circles highlight tetramer formation. CTYO, cytoplasm; Max, maximum; Min, minimum; NUC, nucleus.

We next asked whether chromatin accessibility regulated the basal STAT3 dimer-to-tetramer transition given that DNA binding was found to be necessary for STAT dimer–dimer interaction. We first employed two drugs, trichostatin A and actinomycin D (Fig. 7a), that are known to disrupt or promote chromatin compaction, respectively^36^,^42^,^43^,^44^,^45^. We imaged live HeLa cells expressing STAT3-GFP and STAT3-mCherry (Fig. 7b,c) to conduct brightness (Fig. 7d) and cpCOMB (Fig. 7e) analysis. Loosening chromatin with trichostatin A (ref. 36) or compacting it with actinoymycin D (ref. 45) had no visible effect on STAT3 oligomerization (Fig. 7d). In contrast, the cpCOMB analysis (Fig. 7e) revealed that loosening chromatin promoted accumulation of a STAT3 tetramer population with a very slow transit time (white dashed circles) while compacting chromatin made STAT3 dimers only exhibit the fast transit time. On the basis of our earlier findings in Fig. 6, we concluded that accessible chromatin was required for DNA binding of STAT3 dimers, an event that itself was necessary for tetramer formation (Fig. 5). These experiments highlight how cpCOMB could reveal subtle changes in STAT3 dimer-to-tetramer transition that were missed in the conventional brightness analysis.

**Figure 8:**
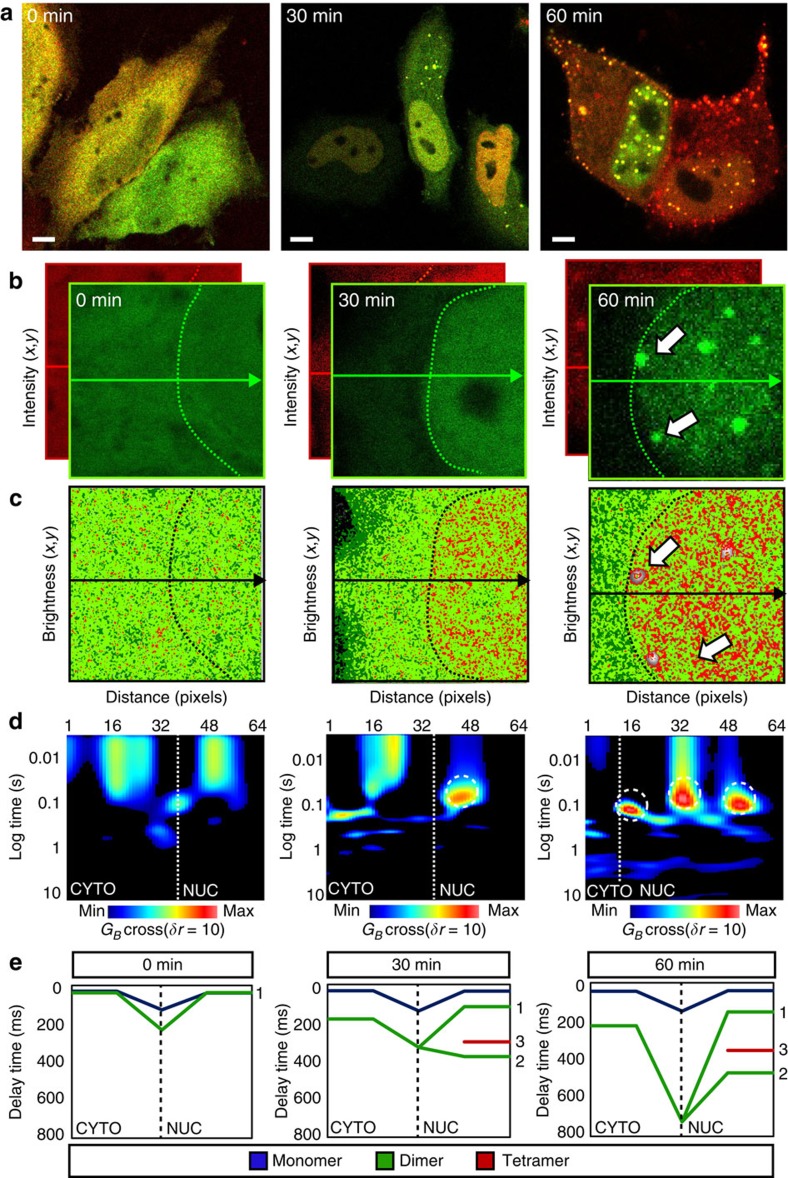
Prolonged cytokine stimulation enhanced STAT3 dimer-to-tetramer transition (cpCOMB analysis). (**a**) HeLa cells transfected with STAT3-GFP and STAT3-mCherry after 0, 30 and 60 min stimulation with 10 nM oncostatin M. Scale bar, 5 μm. (**b**) Intensity images of STAT3-GFP and STAT3-mCherry at 0, 30 and 60 min for a region that spans cytoplasm and nucleus. The nuclear boundary is indicated by the green dotted line. (**c**) Pseudocoloured brightness maps for STAT3-GFP at 0, 30 and 60 min. Dark green pixels represent monomers, light green pixels dimers and red pixels tetramer. At the 60 min time point, the white arrows point to STAT3 puncta that were immobile and so no brightness values were obtained. (**d**) cpCOMB carpets at 0, 30 and 60 min. White dashed circles highlight tetramer formation. (**e**) Transit time for STAT3 to traverse the cytoplasm, nuclear envelope and nucleus as a monomer (blue line), dimer (green line) and tetramer (red line) before and after (30–60 min) oncostatin M stimulation (*n*=6 cells at each time point). After stimulation, a subset of STAT3 dimers diffused freely (1), became immobilized (2) and form immobilized tetramers (3).

To amplify how the STAT3 dimer-to-tetramer transition is regulated during transcriptional activation, we next conducted cpCOMB analysis in HeLa cells expressing STAT3-GFP and STAT3-mCherry, before and after prolonged stimulation with oncostatin M (Fig. 8a). STAT3-GFP and STAT3-mCherry were initially equally distributed throughout the cytoplasm and nucleus (Fig. 8b, 0 min), but stimulation with oncostatin M induced STAT3 nuclear accumulation (Fig. 8b, 30 min) followed by the formation of STAT3 puncta (white arrows in Fig. 8b, 60 min). Brightness analysis of STAT3-GFP over this same time course showed that the initial homogeneous distribution of monomers and dimers throughout the cytoplasm and nucleus of the unstimulated cell (Fig. 8c, 0 min) rearranged to result in the nuclear formation of STAT3 tetramers (Fig. 8c, 30 min). Some STAT3 puncta (Fig. 8c, 60 min) did not give rise to a brightness value due to a lack of fluctuation in intensity at that location, suggesting that they were immobile throughout the data acquisition. This is in agreement with a FRAP study that investigated the dynamics of STAT3 at these puncta and found that cytokine stimulation induced an increasing immobile STAT3 fraction during formation of these nuclear bodies^46^.

By applying pCOMB and cpCOMB analysis in both the cytoplasm–nucleus and nucleus–cytoplasm direction (Fig. 8d and Supplementary Fig. 9a,b, respectively) we found that before stimulation STAT3 monomers and dimers bi-directionally translocate across the nuclear envelope on a timescale of ∼200 ms (Fig. 8e, 0 min, *N*=6 cells). Then, on cytokine stimulation when STAT3 accumulation in the nucleus was observed, STAT3 dimer transport across the nuclear envelope remained bi-directional (albeit on a slower timescale of ∼300 ms) and the intra-nuclear dimer–tetramer translocation dynamics detected in Fig. 6 was reaffirmed (Fig. 8e, 30 min, *N*=6 cells). After prolonged cytokine exposure when STAT3 puncta had formed, STAT3 dimers entered the nucleus on an even more delayed timescale of ∼700 ms, and the intra-nuclear dimer–tetramer transition was promoted in a similar manner to what was observed on loosening of chromatin, with trichostatin A (Fig. 8e, 60 min, *N*=6 cells). Interestingly, a more detailed analysis of the cpCOMB carpet at this time point revealed that nuclear STAT3 tetramers and dimers adopted alternating localizations (Supplementary Fig. 9c), with the STAT3 tetramer mobility always being slightly faster than the STAT3 dimer mobility (Supplementary Fig. 9d).

## Discussion

Understanding how transcription factors employ oligomerization to maintain the many differentiation programs present in metazoans relies on a method that can probe protein mobility as a function of protein stoichiometry *in vivo*, with high spatiotemporal resolution. Here we present such a method that is based on pair correlation analysis of molecular brightness fluctuations acquired along a confocal line scan from a fluorescently labelled transcription factor. We demonstrate that pCOMB analysis can extract the diffusive route of higher-order oligomers in the presence of monomers by first, selectively amplifying the signal from the brightest species present, and second, filtering the dynamics of the extracted oligomeric population based on arrival time between two locations. Extending the pCOMB approach to a two-channel experiment, cpCOMB of a dually labelled fluorescent transcription factor can not only reveal the translocation path of higher-order complexes (here tetramers) but also distinguishes homo- from hetero-complexes and higher-order complexes from sub-complexes (here tetramers from dimers).

We applied the pCOMB and cpCOMB methods to STAT3 and examined the relationship of this transcription factor's oligomeric state with nuclear entry and DNA binding. In particular, we compared wild-type STAT3 with different mutants that inhibit oligomerization^5^, DNA binding^38^ or phosphorylation^40^, by co-expressing both STAT3 variants in the same cytokine-stimulated cell. From these experiments we found that during transcriptional activation STAT3 dimers bi-directionally traverse the nuclear envelope. Inside the nucleus, these dimers either translocate the nuclear space or become immobilized through interactions with DNA. Deletion of the N terminus had no impact on STAT3 nuclear accessibility or intra-nuclear mobility of dimers. However, dimers were required to be phosphorylated and bind DNA in order for tetramer formation. Chromatin accessibility regulated STAT3 oligomerization in the nucleus, suggesting the DNA-bound dimer–dimer interaction is regulated by DNA template access. Prolonged cytokine stimulation resulted in STAT3 nuclear puncta being formed via enhancement of the DNA-bound dimer-to-tetramer transition. Interestingly, in the conditions we examined tetramers had a distinct transit time from DNA-bound dimers and localized at different regions within the nucleus. We concluded that scenario II in Fig. 1a applied, in which tetramer formation is not part of the STAT3 target search strategy. Our findings are underpinned by ChipSeq data reporting STAT3 dimers to bind adjacent GAS elements, which can further interact to form STAT3 tetramers that bend the DNA into a conformation that amplified STAT3 dimer-regulated transcription^6^. Alternatively, STAT3 tetramers may serve as transcriptional repressors^47^, as has been recently postulated for STAT5 (a close analogue of STAT3)^7^,^48^,^49^,^50^, which bind neighbouring genes to inhibit STAT3 dimer transcription.

In summary, we report a new quantitative method that can unravel how oligomerization modulates transcription factor transport and DNA-binding dynamics. Further, because pCOMB and cpCOMB distinguish the translocation of homo- from hetero-complexes and higher-order complexes from sub-complexes, it can also be employed to investigate how complex formation affects intracellular transport, for example, through the nuclear pore complex. This means that one can investigate how protein complex formation modulates access and subsequent interaction with intracellular structures. For example, proteins that employ homo- and/or hetero-oligomerization as a control of their function can now be characterized in the context of the local intracellular architecture. This is particularly important for understanding transcription factor target search, the kinetics of which is greatly influenced by the exploration geometry imparted by chromatin organization^51^,^52^,^53^,^54^. Thus, the pCOMB and cpCOMB methods provide us with a tool to link transcription factor complex stoichiometry to dynamics that may also be extended to other protein complexes.

## Methods

### Cells

HeLa cells (CCL-2, American Type Culture Collection) were grown in high-glucose medium from Invitrogen, supplemented with 10% fetal bovine serum, 5 ml of Pen-Strep and HEPES at 37 °C and in 5% CO_2_. Freshly split cells were plated onto 35-mm glass-bottom dishes coated with fibronectin and then, after 24 h, transiently transfected and co-transfected with the following plasmids: GFP, 2GFP and 5GFP (purchased from Euroscarf); STAT3-GFP, STAT3-DB-GFP and STAT3-YF-GFP (cloned by Ivan Ng in the laboratory of Marie Bogoyevitch and David Jans); STAT3-mCherry (cloned by Zhengmin Yang in the laboratory of Katharina Gaus); and STAT3-YFP and STAT3-NTD-YFP (kindly provided by the laboratory of Gerhard Muller-Newen). Transient transfection was carried out with Lipofectamine 3000 according to the manufacturer's protocol. Stimulation of wild-type and mutant STAT3 activity was carried out by treating the HeLa cells with oncostatin M (10 nM, Sigma Aldrich) for 15 min. STAT3 activation was assessed by the degree of nuclear accumulation. Loosening of chromatin was carried out by treating the HeLa cells transiently transfected with STAT3-GFP and STAT3-mCherry, with trichostatin A (400 nM, Abcam) for 18 h. Compacting of chromatin was carried out by treating the HeLa cells transiently transfected with STAT3-GFP and STAT3-mCherry, with actinomycin D (5 μg ml^−1^, a concentration known to stop class III transcription, Sigma Aldrich) for 30 min. The compaction status of chromatin was assessed by staining the DNA of HeLa cells with Hoechst 33342 (1 μg ml^−1^, Sigma Aldrich) 15 min before imaging. In all line- and frame-scan experiments, cells exhibiting a low STAT3 fluorescent construct expression level were selected, as both the brightness and pCOMB methods are fluctuation-based analyses. Under this selection criteria, variations in STAT3-mCherry expression levels had negligible impact on STAT3 oligomerization (Supplementary Fig. 10).

### Simulations

Two sets of simulations were performed to verify the capability of the pCOMB analysis to resolve the dynamics of different oligomers. In one simulation, monomer and tetramer populations were set to diffuse freely in a two-dimensional 4-μm square box, with the diffusion coefficients equal to 1 μm^2^ s^−1^. In all, 100 particles were used in each simulation, and the fraction of tetramer was varied from 10 to 90% of this population. When the particles exited either boundary of the box, they were re-integrated within the box by the periodic boundary condition. The intensity carpet was produced by convolution of a moving Gaussian beam (full-width at half-maximum=0.2 μm) across the central 64 pixels (3.2 μm). The pixel dwell time was set to 1.56 μs, and its intensity was a convolution of the beam with the particles positions integrated over this time period. At the end of the 64-pixel line, the beam was repositioned back to the first pixel, and the next line was scanned in a similar fashion. The total of 128,000 lines were generated in this way, producing the intensity carpet. To mimic different oligomerization states, a multiplicative factor of 1 and 4 was applied to the convolution step, for a monomer and tetramer, respectively. Analogous simulations were set-up for an increasing fraction of dimers in the presence of monomers and variation of the oligomeric population's diffusion coefficient. All of these simulations were generated in SimFCS from the Laboratory for Fluorescence Dynamics ( www.lfd.uci.edu).

For the nuclear translocation simulation, a semi-permeable barrier was placed at one-third of the simulation box mimicking the nuclear envelope. To the right of the barrier, a uniformly randomly semi-permeable discs were placed to simulate the chromatin compartments. The discs were 0.75 μm in radius at surface density of 0.5 for every μm^2^. The probabilities for dimers of crossing to the right or to the left of the nuclear membrane were set to 0.7 and 0.3, respectively. This ensured that dimer was effectively partitioning and accumulated inside the nuclear (to the right of the barrier) region. The monomer particles were not allowed to diffuse into the nuclear region, while tetramer particles were not allowed to leave the nucleus (translocate to the left of the barrier). Tetramer particles were entering and leaving chromatin compartments with probabilities of 0.9 and 0.1, respectively. This ensured that tetramer was effectively accumulated inside the chromatin-like compartments. The particle densities were set such that their relative ratio was 50:40:10 for monomer: dimer: tetramer. Finally, the diffusion coefficients were set to 5, 3 and 1 μm^2^ s^−1^ for monomer, dimer and tetramer species, respectively. The periodic boundary conditions were applied along the *y* axis, but hard boundary (reflective) was used on left and right edges of the simulation box. This was imposed to avoid that monomers appear inside (right compartment) or tetramer outside (left compartment) of the nucleus. The intensity carpets were generated from the particles' positions using the same approach as described above, and the scanning line was selected to be at least 30 pixels away from either edge, to avoid the potential artefacts in the particles' motion due to the boundary conditions. All of these simulations were generated by a routine written in Matlab.

For the two-colour simulations monomers were assigned either a green or a red channel with 50:50 ratio. Dimers were split into green+green (GG), red+red (RR) and green+red (GR) with 25:25:50 ratios. Finally, tetramers were seeded with four greens (GGGG), four reds (RRRR), three greens+one red (GGGR), three reds+one green (RRRG) and two greens+two reds (GGRR) at 5:5:20:20:50 ratio. The density was set to 3, 1.5 and 0.5 particles per μm^2^ for monomer, dimer and tetramer, respectively. Their diffusion rates were set to 6, 4 and 2 μm^2^ s^−1^, respectively. All of these simulations were generated by a routine written in Matlab.

### Fluorescence microscopy

The microscopy measurements were performed on a Zeiss LSM780 Quasar laser scanning microscope, using a × 40 water immersion objective, 1.2 numerical aperture (Zeiss, Germany). STAT3 constructs fluorescently labelled with GFP, YFP or mCherry were excited with the 488 nm emission line of an Argon laser, 514 nm emission of the Argon laser and the 561 nm emission line of a diode pump solid state laser, respectively. For single (Fig. 3) and sequential two-channel experiments (Figs 4 and 5 and Supplementary Fig. 7), where only pCOMB analysis was performed, STAT3 constructs fluorescently labelled with EGFP, EYFP or mCherry were detected by the internal GaAsP photodetectors between 510 and 560, 520 and 570, and 600 and 600 nm, respectively. For two-channel experiments (Figs 6, 7, 8), where cpCOMB analysis was also performed, the same excitation and detection conditions listed for EGFP and mCherry were used, but in simultaneous acquisition mode. The degree of spectral crosstalk between the two channels was found to be negligible (Supplementary Fig. 8d–e). Image acquisition for number and brightness (N&B) analysis involved selecting a region of interest within a HeLa cell nucleus that vertically placed the nuclear envelope in the middle of the region, at an electronic zoom that resulted in a pixel size of 50 nm for a 256 × 256-pixel frame size. A time series of 100 frames was then collected in the GFP, YFP or mCherry channel at this zoom, with the pixel dwell time set to 12.61 μs, which resulted in a line time of 7.56 ms and a frame time of 1.15 s. Line-scan acquisition for pCOMB and cpCOMB analysis involved selecting a 12.8-μm line along the middle of the N&B image acquisition (perpendicular to the nuclear envelope) and then rapidly scanning this line 1 × 10^5^ times in one or two channels at maximum speed (pixel dwell time 6.3 μs and line time 0.945 ms), with fluorescence being sampled every 200 nm (64 pixels to a line). Calibration of the monomeric brightness and pCOMB amplitude of each fluorescently labelled STAT3 construct tested was performed by the measurement of cells transfected with free GFP, YFP or mCherry under identical frame- and line-scan experimental conditions (Supplementary Figs 3, 4 and 6). FRET interaction between STAT3 constructs was found to have no measurable impact on the brightness distributions detected (Supplementary Fig. 8f).

### Brightness analysis

Brightness analysis of frame- and line-scan acquisitions was performed using a moment analysis described in previously published papers^22^,^23^,^24^. Briefly, in each pixel of a line scan we have a temporal intensity fluctuation that has a temporal average 〈*F*(*t*)〉 (first moment) and variance 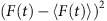 (second moment). The ratio of these two properties describes the apparent brightness (B) of the molecules that give rise to the intensity fluctuation, as described in the following equation:


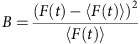


In the case of a photon counting detector, the true molecular brightness (*ɛ*) of the molecules are related to the measured apparent brightness (*B*) by *B*=*ɛ*+1, where 1 is the brightness contribution of the detector given that the photon-counting detector variance (*σ*^2^_detector_) should be equal to the average intensity of the detector noise 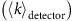. In the case of an analog detector this is not true due to characteristics of the analog amplifier and the settings of the analog-to-digital converter. Thus, the detector's brightness contribution needs to be accounted for by a term called the *S* factor, which returns the background brightness to 1 so that the molecular brightness of the molecules can be extracted. The number of intensity time points used for each moment calculation needs to contain a sufficient number of fluctuations to ensure good statistics and span over a temporal window that allows the dynamics being probed to decay. Depending on the width of the temporal window and the sampling frequency of the acquisition, the analysis is tuned to detect different timescales of mobility. Here we use 100 points to derive each brightness value in the frame and line scans. This gives rise to a single brightness map for the image acquisition. In the case of the line scans (which contain 10^5^ lines), we shift the 100 time points window by a single line, and for every window position calculate *B* to obtain a brightness carpet. It is represented by the *x* coordinate corresponding to the point along the line (pixels), the *y* coordinate corresponding to the time of acquisition and the pseudocolour indicates the local effective brightness value. All brightness calculations were made by a routine written in Matlab and can also be carried out from the scanning FCS page in SimFCS from the Laboratory for Fluorescence Dynamics ( www.lfd.uci.edu).

### Pair correlation analysis of brightness fluctuations

Pair correlation analysis of the derived brightness fluctuations along each line-scan acquisition was performed using the following function that is adapted from previously published papers^26^,^27^,^28^,^37^:


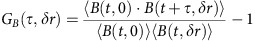


The pair correlation brightness function is displayed in pseudocolours in an image in which the *x* coordinate corresponds to the pixel position along the line and the *y* coordinate corresponds to the correlation time in a log scale. Because the amplitude of each pair correlation function is proportional to the weighted square of the brightness of the molecules that translocate from that location, the amplitude for monomeric GFP, YFP or mCherry molecular flow must first be calibrated so that oligomeric translocations can be assigned (Supplementary Figs 3, 4 and 6). Pair correlation amplitude maxima were thus extracted from each column and plotted as function of pixel position, for a given laser power, detector gain and pair correlation distance (*δr*). The average value of the amplitude maxima along the calibration line was set as the threshold for monomer versus oligomer translocation. The extrapolated amplitude maxima value for higher-order oligomer translocation was expected to range from *G*_monomer_ to *G*_monomer_ × oligomeric state^2^. In general the pair correlation distance (*δr*) was set to 6–10 pixels, as these conditions tested molecular flow across the cytoplasm, across the nuclear envelope and within the nucleus. All pair correlation calculations were made by a routine written in Matlab and can also be carried out from the scanning FCS page in SimFCS from the Laboratory for Fluorescence Dynamics ( www.lfd.uci.edu).

### Cross-pair correlation analysis of brightness fluctuations

Cross-pair correlation analysis of the derived brightness fluctuations along each line-scan acquisition was performed using the following function that is adapted from previously published papers^27^:


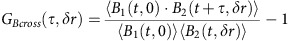


The cross-pair correlation brightness function is displayed in pseudocolours in an image in which the *x* coordinate corresponds to the pixel position along the line and the *y* coordinate corresponds to the correlation time in a log scale. The amplitude of each cross-pair correlation function is proportional to the weighted square of the brightness of the molecules that translocate from that location in channel 1 with respect to channel 2. Thus, the correlation calibrations carried out for monomeric GFP and mCherry molecular flow can be used to predict the correlation amplitude for heterodimer translocation. All cross-pair correlation calculations were made by a routine written in Matlab and can also be carried out from the scanning FCS page in SimFCS from the Laboratory for Fluorescence Dynamics ( www.lfd.uci.edu).

## Additional information

**How to cite this article:** Hinde, E. *et al*. Quantifying the dynamics of the oligomeric transcription factor STAT3 by pair correlation of molecular brightness. *Nat. Commun.* 7:11047 doi: 10.1038/ncomms11047 (2016).

## Supplementary Material

Supplementary Information Supplementary Figures 1-10
