# Supplementary material for: Quantifying the dynamics of the oligomeric transcription factor STAT3 by pair correlation of molecular brightness
Source: Nat Commun. 2016 Mar 24;7:11047. doi: 10.1038/ncomms11047 (PMC4820838; doi:10.1038/ncomms11047)
Supplement: Supplementary Information — Supplementary Figures 1-10 [file ncomms11047-s1.pdf]

SUPPLEMENTARY FIGURES

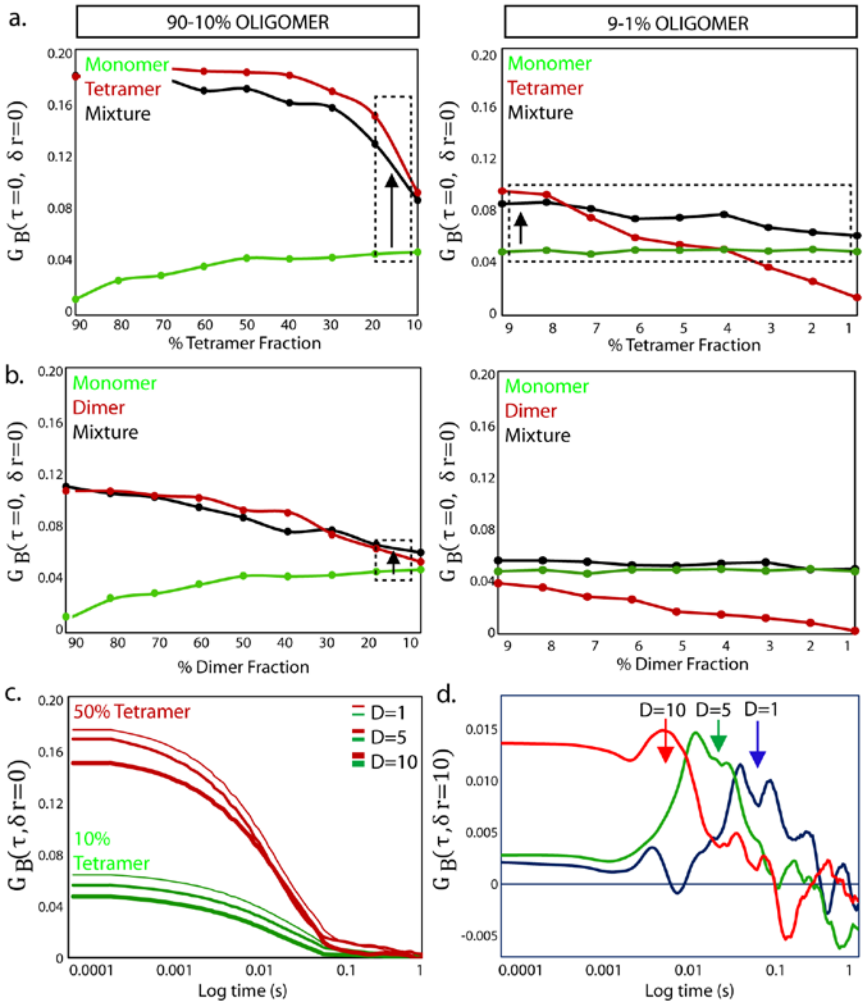

Supplementary Figure 1. Rules of pCOMB analysis. Simulation of low levels of tetramer and dimer (1-9%) in the presence of monomers (extension of **Fig. 2**). (**a)** In **Fig. 2** we found from auto-correlation analysis of mixed populations of tetramers and monomers, that tetramer fractions as low as 10-20% (black arrow) are easily distinguishable from monomers based on correlation amplitude ($G_B(0)$). Extension of this simulation to a tetramer fraction of 9-1% (a percentage that is relevant to the fraction of transcription factor bound to DNA at any time) demonstrates that this distinction is maintained and the $G_B(0)$ value for the mixed population remains sufficiently different to the $G_B(0)$ value for monomeric molecular flow. **(b)** Auto-correlation analysis of mixed populations of dimers and monomers demonstrates that dimer fractions as low as 10-20% (black arrow) are not as easily distinguishable from monomers based on correlation amplitude ($G_B(0)$) (unlike tetramers). Extension of this simulation to a dimer fraction of 9-1% demonstrates that distinction of low levels of dimers from monomers based on $G_B(0)$) is not possible, as the mixed population has an identical $G_B(0)$ value to monomeric molecular flow. **(c-d)** Auto- (δr=0, **c**) and pair- (δr=10, **d**) correlation analysis of simulations where the tetramers are diffusing with a coefficient of 1, 5 and 10 $\mu m^2 s^{-1}$ illustrates that the diffusion coefficient does not significantly affect autocorrelation amplitude (as compared to the fraction of oligomer) but rather the peak time of the pair correlation function.

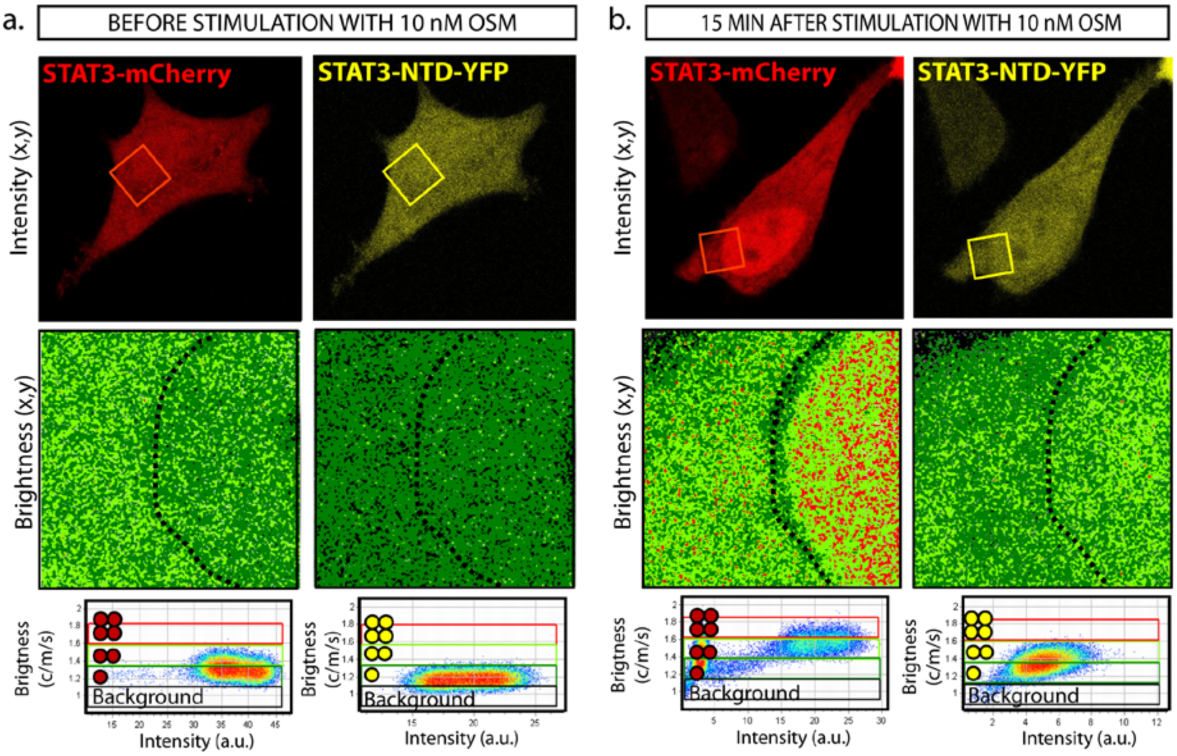

Supplementary Figure 2. Brightness analysis of STAT3-NTD-YFP mutant before and after 15 min of Oncostatin M treatment. (a) Brightness analysis of a HeLa cell co-transfected with STAT3-mCherry and STAT3-NTD-YFP before treatment with 10 nM of Oncostatin: dark green pixels represent monomers, light green pixels dimers and red pixels tetramers. In resting cells, the STAT3-NTD-YFP mutant existed as a purely monomeric population. (**b**) Brightness analysis of a HeLa cell co-transfected with STAT3-mCherry and STAT3-NTD-YFP 15 min after treatment with 10 nM of Oncostatin. In stimulated cells the STAT3-NTD-YFP mutant formed a dimeric population, but was unable to further dimerise into tetramers. Calibration of the monomeric brightness of free mCherry and EYFP is presented in **Supplementary Figure 3a-d** and **Supplementary Figure 4a-d**, respectively.

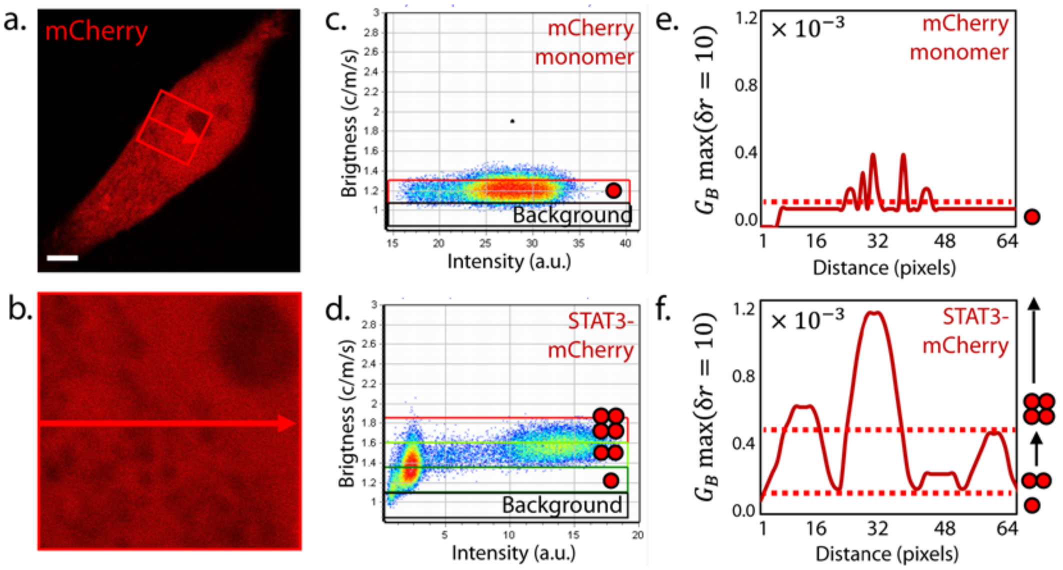

Supplementary Figure 3. Calibration of monomeric mCherry brightness and pCOMB amplitude. (a) HeLa cell transfected with monomeric mCherry. Scale bar = 5 µm. (**b**) Intensity image of the region of interest selected for calibration of monomeric mCherry brightness and the position of the line scan acquisition used for calibration of monomeric mCherry molecular flow superimposed. (**c**) Brightness analysis of monomeric mCherry. (**d**) Brightness analysis of STAT3-mCherry using the monomeric mCherry calibration finds an apparent brightness distribution that extends from monomers to tetramers (corresponding brightness map is presented in **Fig. 4b**). (**e**) pCOMB maximum amplitude of monomeric mCherry (G_{Bmax}) in each pixel along line scan when δr=10. Dashed line represents the average pCOMB amplitude and this value was used to calibrate higher order oligomer translocation. (f) Analysis of pCOMB maximum amplitude for STAT3-mCherry using the monomeric mCherry calibration, reveals molecular flow of STAT3-mCherry dimers and tetramers (corresponding pCOMB carpet presented is presented in **Fig. 4e**).

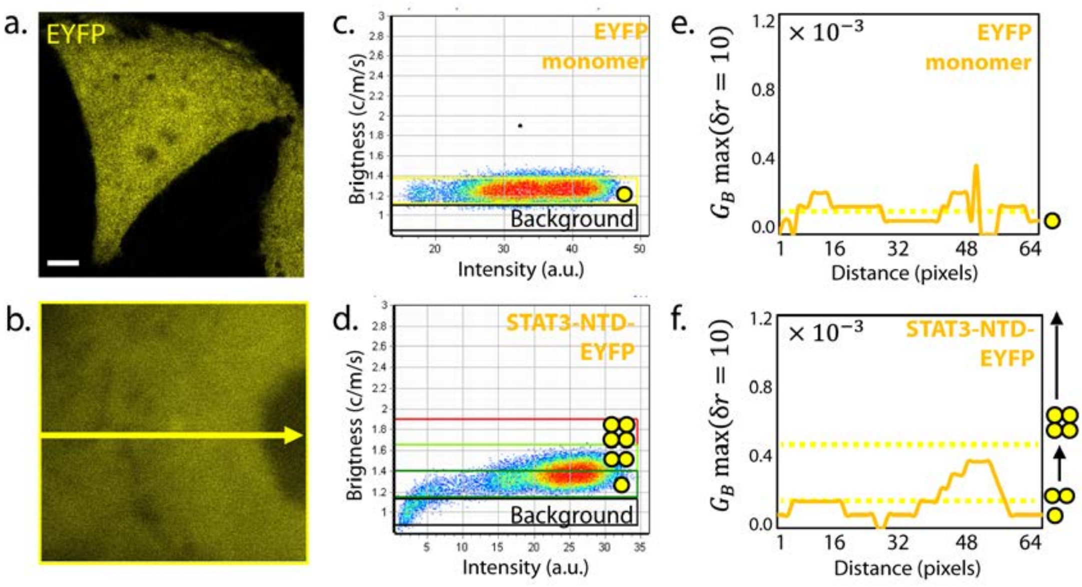

Supplementary Figure 4. Calibration of monomeric EYFP brightness and pCOMB amplitude. (a) HeLa cell transfected with monomeric EYFP. Scale bar = 5 µm. (**b**) Intensity image of the region of interest selected for calibration of monomeric EYFP brightness, with the position of the line scan acquisition used for calibration of monomeric mCherry molecular flow superimposed. (**c**) Brightness analysis of monomeric EYFP. (**d**) Brightness analysis of STAT3-NTD-EYFP using the monomeric EYFP calibration finds an apparent brightness distribution that extends from monomers to dimers (corresponding brightness map is presented in **Fig. 4f**). (**e**) pCOMB maximum amplitude of monomeric EYFP (G_{Bmax}) in each pixel along line scan when δr=10. Dashed line represents the average pCOMB amplitude and this value was used to calibrate higher order oligomer translocation. (**f**) Analysis of pCOMB maximum amplitude for STAT3-NTD-EYFP using the monomeric EYFP calibration, reveals molecular flow of STAT3-NTD-EYFP monomers and dimers (corresponding pCOMB carpet presented is presented in **Fig. 4i**).

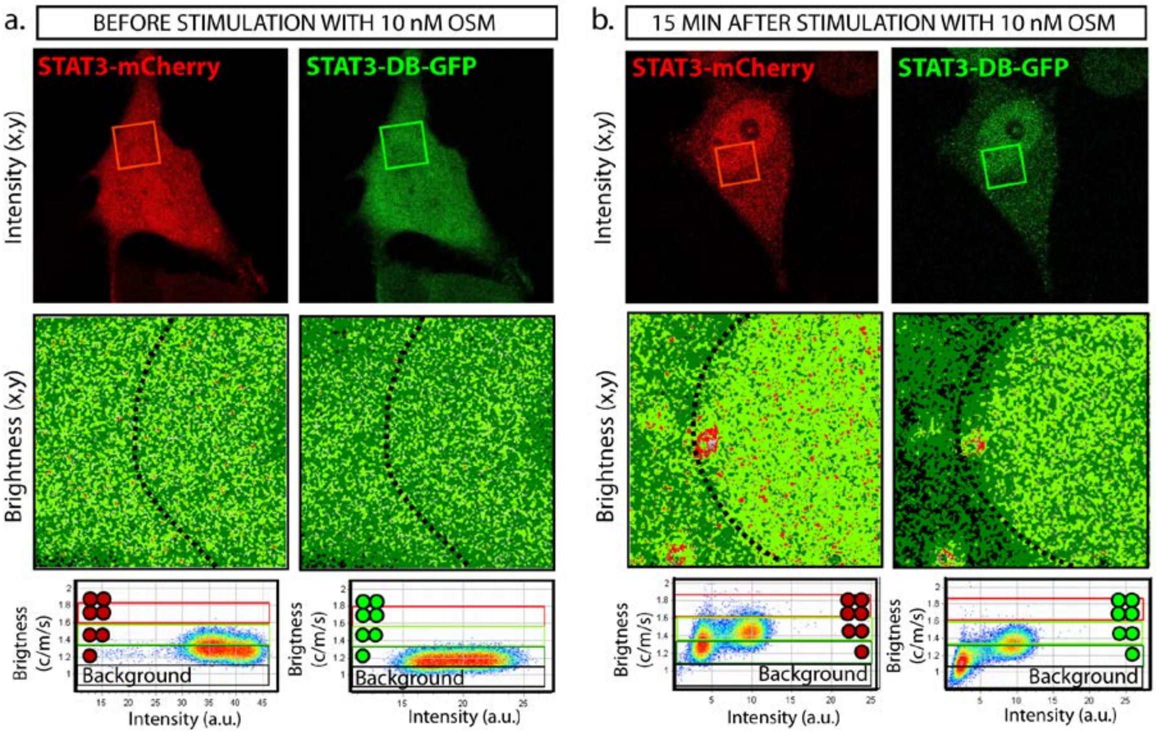

Supplementary Figure 5. Brightness analysis of STAT3-DB-GFP mutant before and after 15 min of Oncostatin M treatment. (a) Brightness analysis of a HeLa cell co-transfected with STAT3-mCherry and STAT3-DB-GFP before treatment with 10 nM of Oncostatin: dark green pixels represent monomers, light green pixels dimers and red pixels tetramers. In resting cells the STAT3-DB-GFP mutant exhibited the same oligomeric subcellular distribution as wild-type STAT3. (**b**) Brightness analysis of a HeLa cell co-transfected with STAT3-mCherry and STAT3-DB-GFP 15 min after treatment with 10 nM of Oncostatin. In stimulated cells the STAT3-DB-GFP mutant inhibited nuclear dimer and tetramer formation. Calibration of the monomeric brightness of free mCherry and EGFP is presented in **Supplementary Figure 3a-d** and **Supplementary Figure 6a-d**, respectively

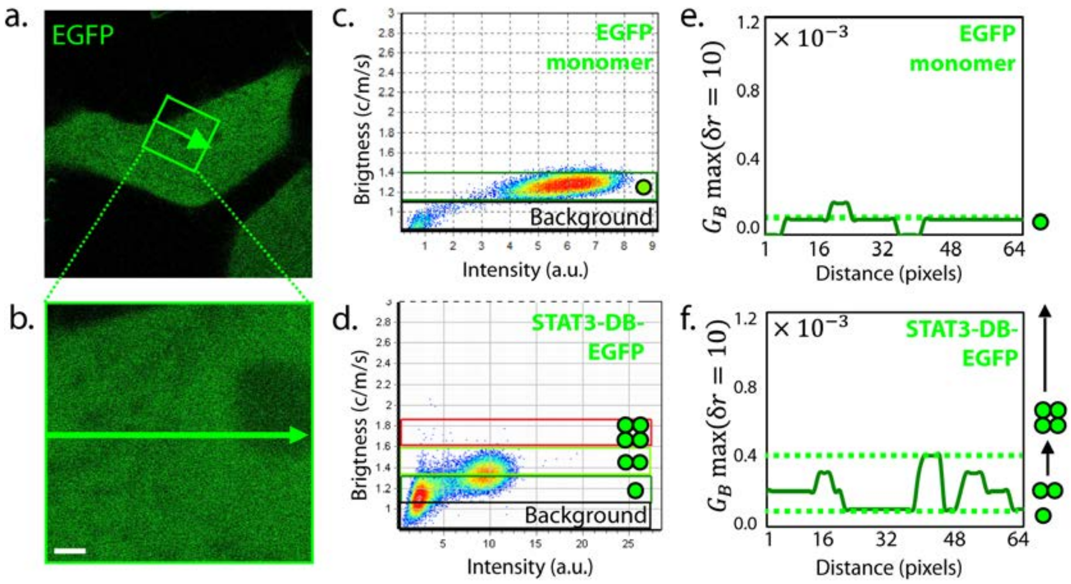

Supplementary Figure 6. Calibration of monomeric EGFP brightness and pCOMB amplitude. (a) HeLa cell transfected with monomeric EGFP. Scale bar = 5 µm. (**b**) Intensity image of the region of interest selected for calibration of monomeric EGFP brightness, with the position of the line scan acquisition used for calibration of monomeric mCherry molecular flow superimposed. (**c**) Brightness analysis of monomeric EGFP. (**d**) Brightness analysis of STAT3-DB-EGFP using the monomeric EGFP calibration finds an apparent brightness distribution that extends from monomers to dimers (corresponding brightness map is presented in **Fig. 5b**). (**e**) pCOMB maximum amplitude of monomeric EGFP (G_{Bmax}) in each pixel along line scan when δr=10. Dashed line represents the average pCOMB amplitude and this value was used to calibrate higher order oligomer translocation. (**f**) Analysis of pCOMB maximum amplitude for STAT3-DB-EGFP using the monomeric EGFP calibration, reveals molecular flow of STAT3-DB-EGFP monomers and dimers (corresponding pCOMB carpet presented is presented in **Fig. 5e**).

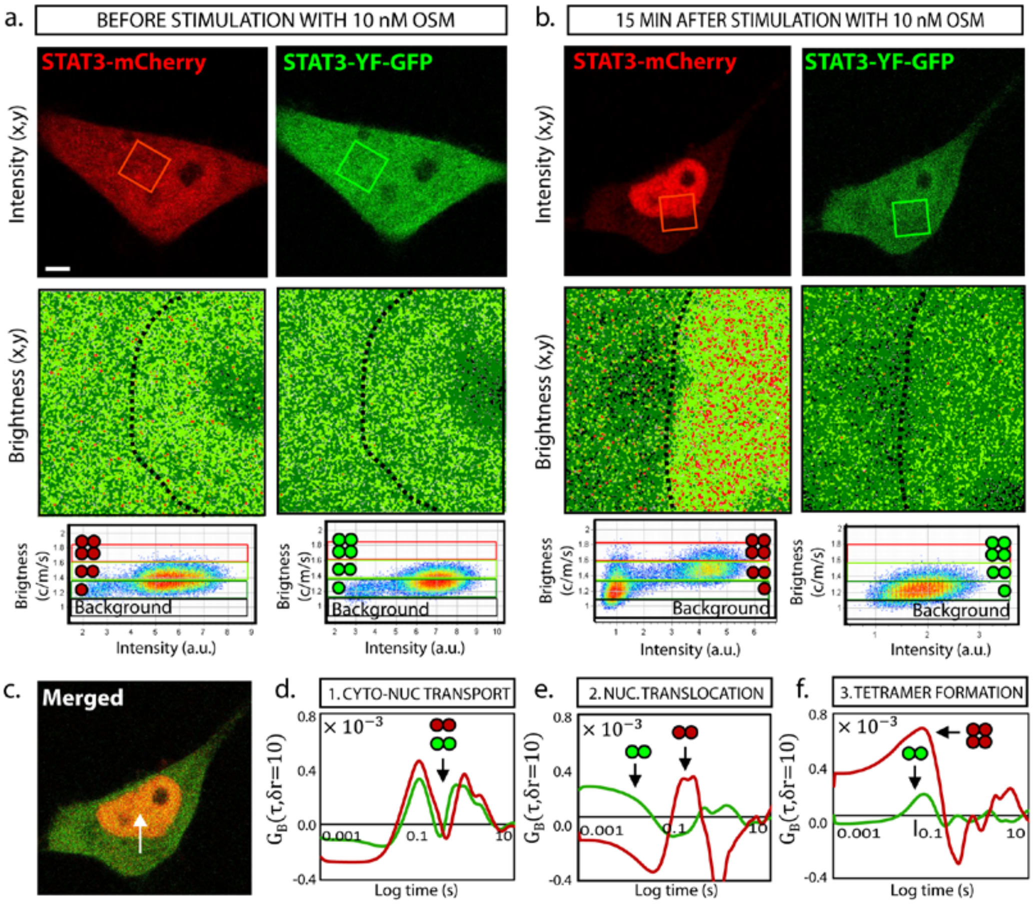

Supplementary Figure 7. Brightness and pCOMB analysis of the STAT3-YF-GFP mutant before and after 15 min of Oncostatin M treatment. (a) Brightness analysis of a HeLa cell co-transfected with STAT3-mCherry and STAT3-YF-GFP before treatment with 10 nM of Oncostatin: dark green pixels represent monomers, light green pixels dimers and red pixels tetramers. In resting cells the STAT3-YF-GFP mutant exhibits the same oligomeric subcellular distribution as wild type STAT3. (**b**) Brightness analysis of a HeLa cell co-transfected with STAT3-mCherry and STAT3-YF-GFP 15 min after treatment with 10 nM of Oncostatin. In stimulated cells the STAT3-YF-GFP mutant was neither able to accumulate in the nucleus nor to dimerise into tetramers. Calibration of the monomeric brightness of free mCherry and EGFP is presented in **Supplementary Figure 3a-d** and **Supplementary Figure 6a-d**, respectively. **(c)** Position of the two-colour line scan acquired across the nuclear envelope of a stimulated HeLa cell (condition as in **b**) co-transfected with STAT3-mCherry and STAT3-YF-GFP for pCOMB analysis. (**d-f**) pCOMB profiles for STAT3-mCherry (red) and STAT3-YF-GFP (green) translocation across the nuclear envelope (**d**), intra-nuclear dimer translocation (**e**) and intra-nuclear tetramer formation (**f**).

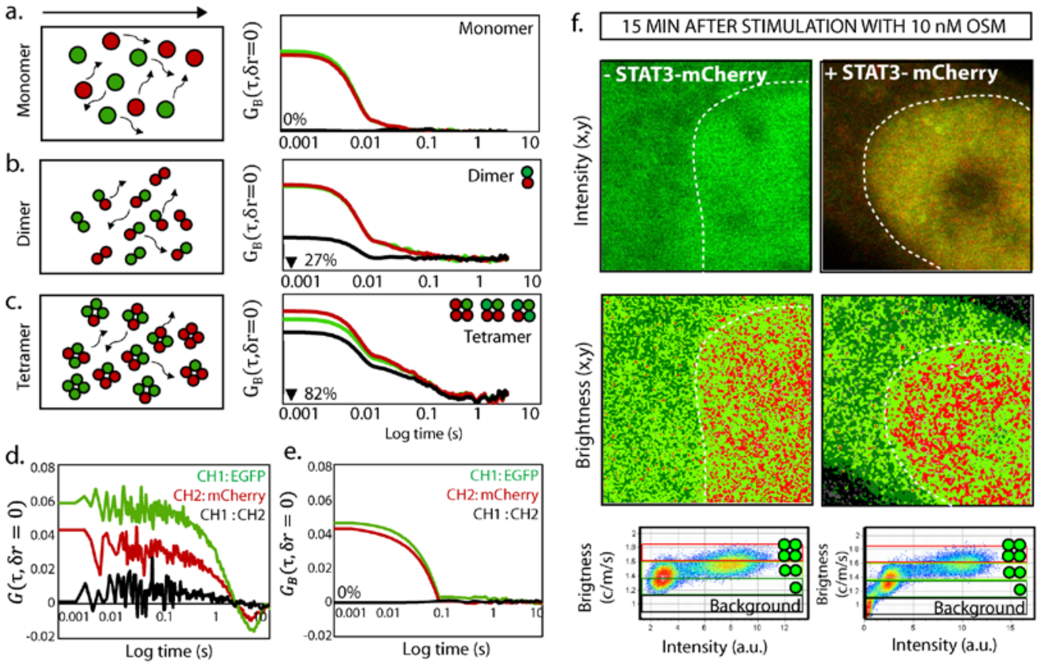

Supplementary Figure 8. Cross pair correlation analysis for dimer detection (a-b), spectral cross-talk between EGFP and mCherry (d-e), and lack of FRET between STAT3-GFP and STAT3-mCherry (f). (**a**) Simulation of a two-channel line scan for a population of green and red monomers results in 0% cross correlation of the brightness fluctuations. (**b**) Simulation for green homo-dimers, red homo-dimers and green-red hetero-dimers resulted in 27% cross correlation of the brightness fluctuations. (**c**) Simulation for green and red homo- and hetero-tetramers resulted in 82% cross correlation of the brightness fluctuations. **(d-e)** Auto- and cross correlation analysis of a two-channel line scan acquisition of free EGFP and free mCherry (see Methods section) revealed negligible spectral cross-talk (0% cross correlation between CH1:CH2) when analysing fluctuations in fluorescence intensity **(d)** and brightness **(e)**. **(f)** Brightness analysis of STAT3-GFP in the absence and presence of STAT3-mCherry in a stimulated HeLa cell (15 min of 10 nM Oncostatin M): dark green pixels represent monomers, light green pixels dimers and red pixels tetramers. Agreement between brightness maps demonstrates FRET interaction between GFP and mCherry upon STAT3 oligomerisation to have negligible impact on the brightness analysis. Calibration of the monomeric brightness of free EGFP is presented in **Supplementary Figure 6a-d**.

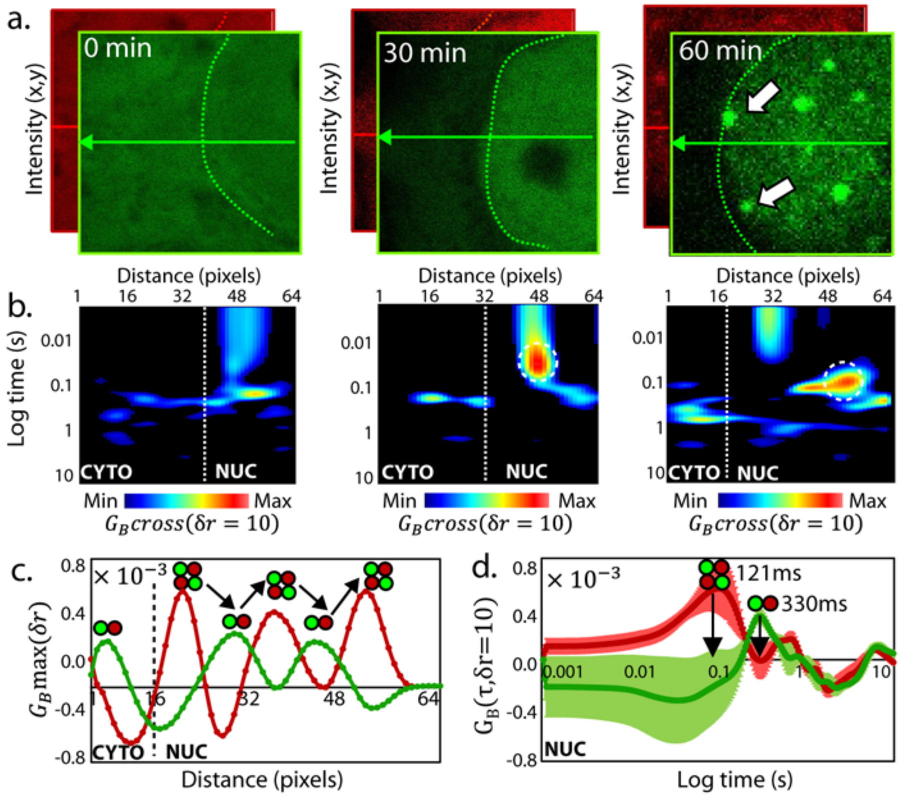

Supplementary Figure 9. Oncostatin M-stimulated STAT3 transport from the nucleus to the cytoplasm (additional cpCOMB analysis for the data presented in Figure 8). (**a**) HeLa cells transfected with STAT3-GFP and STAT3-mCherry at 0 min, 30 min and 60 min after stimulation with 10 nM Oncostatin M. Green arrow indicates scan direction. White arrows at 60 min point to STAT3 puncta. Scale bar = 5 µm. (**b**) cpCOMB carpets at 0 min, 30 min and 60 min testing translocation from the nucleus (CYTO) to the cytoplasm (NUC). White dashed circles indicate tetramer formation. **(c)** cpCOMB amplitude along the scanned line at 60 min from the cytoplasm (CYTO) to nucleus (NUC) revealed tetramers and dimers to adopted alternating localisations within the nucleus. (**d**) cpCOMB analysis of the nuclear translocation time for these two species at 60 min was 121 ms for tetramers and 330 ms for dimers.

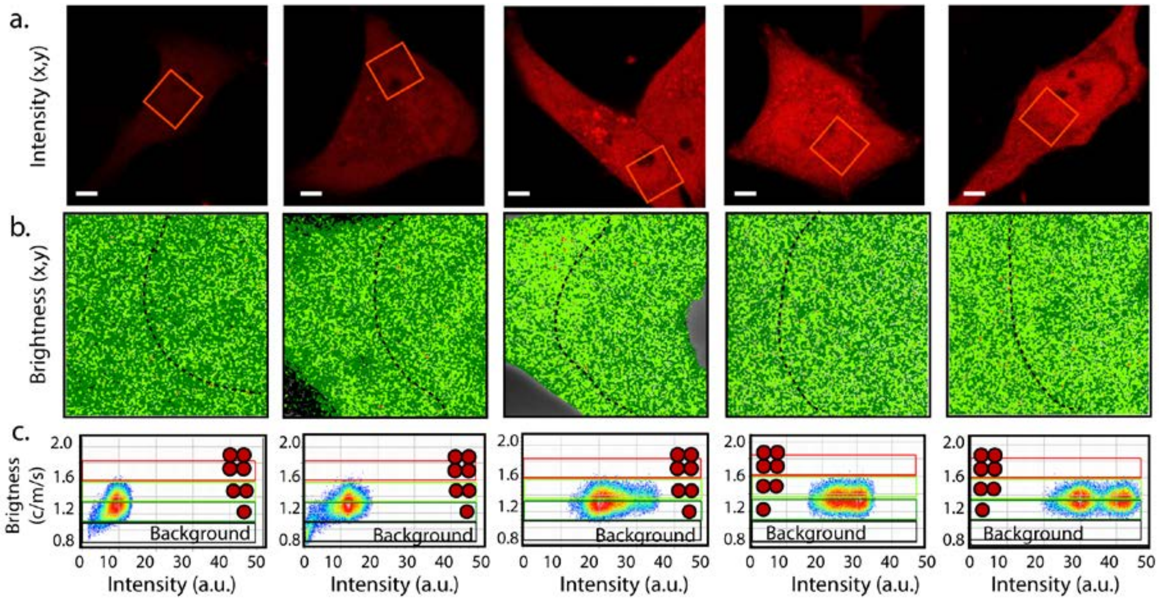

Supplementary Figure 10. Brightness analysis to determine the impact of STAT3-mCherry expression level on STAT3 oligomerisation in HeLa cells. (a) STAT3-mCherry fluorescence intensity in five different HeLa cells with increasing expression level. **(b-c)** Brightness analysis of STAT3-mCherry in the five different HeLa cells reveals that the oligomeric sub-cellular distributions **(b)** and brightness histograms **(c)** was not significantly affected by differences in STAT3-mCherry concentration. Calibration of the monomeric brightness of free mCherry is presented in **Supplementary Fig. 3a-d**.
